# Cortical dynamics during cell motility are regulated by CRL3^KLHL21^ E3 ubiquitin ligase

**DOI:** 10.1038/ncomms12810

**Published:** 2016-09-19

**Authors:** Thibault Courtheoux, Radoslav I. Enchev, Fabienne Lampert, Juan Gerez, Jochen Beck, Paola Picotti, Izabela Sumara, Matthias Peter

**Affiliations:** 1 Institute of Biochemistry, Department of Biology, ETH Zurich, Otto-Stern-Weg 3, 8093 Zürich, Switzerland; 2 Institut de Génétique et de Biologie Moléculaire et Cellulaire (IGBMC), Centre National de la Recherche Scientifique UMR 7104, Institut National de la Santé et de la Recherche Médicale U964, Université de Strasbourg, 67404 Illkirch, France

## Abstract

Directed cell movement involves spatial and temporal regulation of the cortical microtubule (Mt) and actin networks to allow focal adhesions (FAs) to assemble at the cell front and disassemble at the rear. Mts are known to associate with FAs, but the mechanisms coordinating their dynamic interactions remain unknown. Here we show that the CRL3^KLHL21^ E3 ubiquitin ligase promotes cell migration by controlling Mt and FA dynamics at the cell cortex. Indeed, KLHL21 localizes to FA structures preferentially at the leading edge, and in complex with Cul3, ubiquitylates EB1 within its microtubule-interacting CH-domain. Cells lacking CRL3^KLHL21^ activity or expressing a non-ubiquitylatable EB1 mutant protein are unable to migrate and exhibit strong defects in FA dynamics, lamellipodia formation and cortical plasticity. Our study thus reveals an important mechanism to regulate cortical dynamics during cell migration that involves ubiquitylation of EB1 at focal adhesions.

## Abstract (web summary)

Although focal adhesions (FAs) and microtubules (MTs) are known to associate, the underlying regulation of this dynamic interaction is not understood. Here the authors discover that the CRL3^KLHL21^ E3 ubiquitin ligase localises to FAs and ubiquitinates the MT plus-tip binding protein EB1, thereby promoting MT and FA dynamics and cell migration.

## Graphical abstract

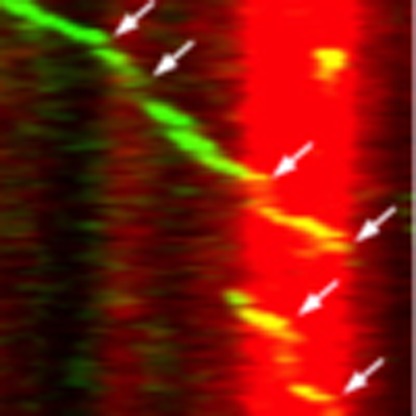

Cell migration is essential for tissue organization and regeneration, and defects in the underlying processes have been associated with many developmental disorders and cancer progression. Directed cell migration requires cell polarization and the coordinated action of the actin and microtubule (Mt) cytoskeletons^1^. However, the spatial and temporal mechanisms that link actin and Mt dynamics are poorly understood.

Cell migration requires sustained forward movement of the plasma membrane at the leading edge. Actin polymerization directly pushes the plasma membrane forward using a combination of actomyosin-based contractility and reversible detachment of membrane from cortical actin cytoskeleton. Dynamic Mts are also required during the migration process^1^,^2^, but their function at the cortex is less clear. Individual Mts are polarized filaments, with plus ends that grow, shrink or pause in a process termed dynamic instability^3^. Mt dynamics are regulated by multiple components including motor proteins and crosslinking factors, as well as by post-transcriptional modifications^4^.

Mt-plus ends are highly dynamic and comprise a loading platform for Mt-plus-end interacting proteins called +TIPs^5^, like the family of end binding (EB) proteins that includes EB1, EB2 and EB3. EB1 forms dimers, that autonomously track Mt tips by recognizing structural motifs on growing Mt ends^6^,^7^,^8^,^9^,^10^. The structure of the EB1 amino-terminal domain, encompassing conserved CH-domain, has been determined in complex with α-β tubulin heterodimers by cryo-electron microscopy^11^. The C-terminal domain of EB1 binds +TIPs partners including the adenomatous polyposis coli (APC) tumour suppressor, the Mt–actin binding protein (MACF), the cytoplasmic linker protein (CLIP170) and Clip-associated proteins (CLASPs)^12^. A conserved SxIP motif in +TIP proteins targets them to Mt-plus ends in an EB1-dependent manner^13^. Indeed, a proteome-wide screen for SxIP-containing proteins has not only identified CLASPs, MACF1 and APC, but also several new +TIP proteins^14^, implying that EB1 interacts with multiple components and thereby contributes to many Mt-dependent functions.

Cell movement requires dynamic interactions of the actin and Mt networks with the extracellular matrix through focal adhesions (FAs)^15^. At the leading edge, integrin-dependent mechanisms promote the assembly of new FA structures, while FAs at the cell rear must be destabilized. CLASPs, MACF1/ACF7 and APC physically link actin and Mts at FAs, and promote cell migration by regulating FA dynamics and establishing directed Mt transport pathways^16^. The scaffolding component Paxillin was shown to function as a local Mt catastrophe factor at FA^17^, and its phosphorylation by the FA kinase (FAK) may regulate FA dynamics^18^. However, the mechanisms by which Mts dynamically interact with FA and promote FA-turnover remain poorly understood.

Protein ubiquitylation triggers both proteasome-dependent and -independent mechanisms that emerged as a wide-spread regulatory signal in cellular physiology including cell signalling, intracellular trafficking and mitosis^19^,^20^. Substrate ubiquitylation requires the coordinated action of three enzymes catalyzing ubiquitin activation (E1), ubiquitin conjugation (E2) and ubiquitin ligation (E3). Cullin-RING E3 ubiquitin ligases (CRLs) comprise the largest class of E3 enzymes, which primarily use the RING-H2 finger protein Rbx1 ubiquitin-charged E2 enzymes and catalyse ubiquitin transfer^21^. CRLs are activated by modification of the cullin subunit with the ubiquitin-like protein Nedd8, which is reversed by the action of the COP9/signalosome complex (CSN)^22^. Specificity is provided by binding of the Cullin scaffold to subfamily-specific adaptors, which in turn recruit substrates into the complex. For example, CRL3 assembles with Bric-a-brac/Tramtrak/Broad (BTB) proteins such as Keap1 to regulate stress signalling by targeting Nrf2 (refs 23, 24), or KLHL12, which promotes collagen secretion by ubiquitylating Sec31 (ref. 25). KLHL21 and KLHL22 have been shown to target Aurora B and PLK1, respectively, thereby regulating chromosome alignment at the metaphase-to-anaphase transition^26^,^27^. Finally, CRL3 recently emerged as a tumour driver and recurrent mutations in the BTB adaptor SPOP may define a new molecular subtype of prostate cancer^28^.

In this study, we identified a new function of CRL3^KLHL21^ as a regulator of cell motility by spatially and temporally regulating Mt dynamics at the cell cortex and at FAs. Nanoscale multi-coloured microscopy on fixed and live samples revealed that KLHL21 transiently localizes to Mt tips and accumulates at FAs, preferentially at the leading edge of migrating cells. CRL3^KLHL21^ binds and ubiquitylates EB1 predominantly on Lys100, which is located within the amino-terminal CH-domain known to interact with Mts. Functional analysis reveals cells expressing a non-ubiquitylatable EB1^K100R^ mutant phenocopy CRL3^KLHL21^ depletion, and are defective for cell migration and FA dynamics. We propose that CRL3^KLHL21^ promotes FA dynamics and cell motility at least in part through ubiquitylation of EB1 at Mts reaching the cell cortex.

## Results

### The CRL3-adaptor protein KLHL21 controls cell migration

**Figure 1:**
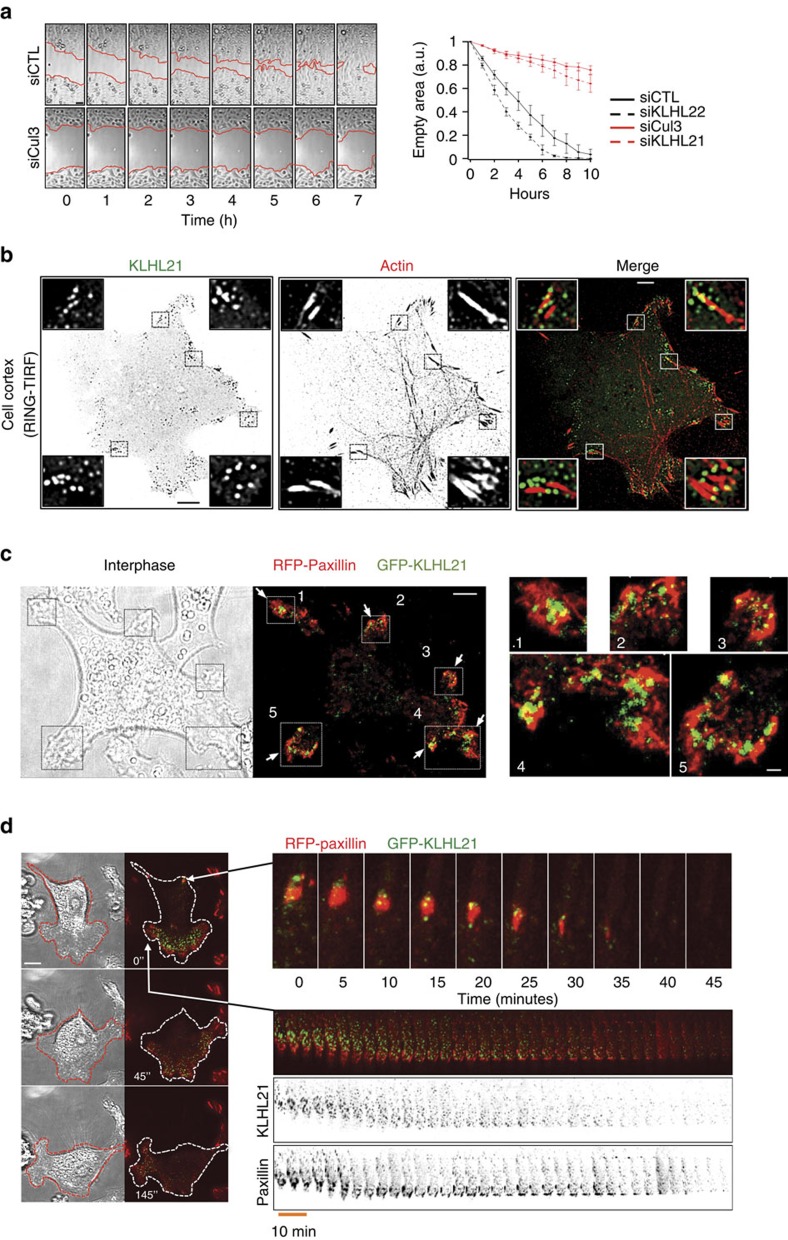
**Cells lacking CRL3**^**KLHL21**^ **exhibit defects in cell migration.** (**a**) Confluent HeLa cultures treated with the indicated siRNA oligos were scratched (time 0) and the resulting cell-free zone (area within red line) was imaged every hour during closure (Supplementary Movie 1). Scale bar, 60 μm. The empty area was quantified for each siRNA condition, and plotted as arbitrary units (a.u.) against the time after scratching (hours). Error bars indicate variability of three independent experiments. (**b**) The cortex of living HeLa-FRT/TO cells stably expressing GFP-KLHL21 (left) and stained with low doses of SIR-actin dye (middle) to mark FA was imaged using RING-TIRF microscopy (Supplementary Movie 2, single plane acquisition). Insets show boxed regions at higher magnification. Scale bar, 5 μm. (**c**) and (**d**) HeLa-FRT/TO cells stably expressing GFP-KLHL21 (green) and transfected with RFP-Paxillin (red) plasmid were observed using RING-TIRF for several hours. Scale bar, 5 μm. (**c**) Immobile cell. White arrows show GFP-KLHL21 localization at FA proximity (RFP-Paxillin). Insets show numbered squares at higher magnification. Scale bar 1 μm, Supplementary Movie 3. (**d**) Mobile cell over time (left panel). Time series of magnified region showing disappearance of FA marker RFP-Paxillin and dynamics of GFP-KLHL21 (upper right panel). Kymograph shows dynamics of a large lamellipodia (lower right panel, Supplementary Movie 4).

To investigate the function of CRL3^KLHL21^ in cytoskeletal regulation, we analysed migration of HeLa cells using a scratch assay. Interestingly, cells depleted for Cul3 or KLHL21 by RNAi were unable to fill the scratch (Fig. 1a; Supplementary Movie 1). To quantify this migration defect, we measured the scratch area during closure over a period of 10 h (Fig. 1a, right panel). In contrast to controls, cells depleted for Cul3 or KLHL21 by RNAi were highly immobile and exhibited cortex deformations resulting in elongated cell morphology (Supplementary Movie 1). This defect was not observed in cells depleted for KLHL22, which like KLHL21 exhibits delays in mitosis^26^,^27^.

To determine how CRL3^KLHL21^ promotes cell migration, we first examined the subcellular localization of KLHL21 in HeLa and U20S cells expressing functional GFP-tagged KLHL21 from the doxycycline (Dox)-inducible promoter at similar levels as endogenous KLHL21 (Supplementary Fig. 1A). We used a new enhanced total internal reflection (TIRF) microscopy called RING-TIRF that reduces limitations of an irregular illumination field and allows near simultaneous acquisition of multiple wavelengths in high-speed mode^29^. We localized GFP-KLHL21 and actin in living cells together on structures close (80–120 nm) to the Hela cell surface (Fig. 1b; Supplementary Movie 2). Moreover, we followed single cells expressing GFP-KLHL21 and RFP-Paxillin or stained for VASP, in which the latter two signals are known to mark FA structures (Fig. 1c,d; Supplementary Fig. 1B). Interestingly, GFP-KLHL21 accumulated at the cell periphery in small, immobile areas that co-localized with actin, Paxillin and VASP (Fig. 1b–d; Supplementary Fig. 1B; Supplementary Movie 2–4). These cortical GFP-KLHL21 patches disappeared upon disassembly of FA sites early in mitosis, but were re-established during cytokinesis (Supplementary Fig. 1C; Supplementary Movie 3). To follow the dynamics during cell migration, we performed RING-TIRF live cell imaging of motile HeLa cells co-expressing GFP-KLHL21 (green) and RFP-Paxillin (red). As shown in Fig. 1d (Supplementary Movie 4), KLHL21 and Paxillin co-localized at FAs at lamelipodia (white arrows, lower right kymograph) and remained throughout their disassembly (white arrows, upper right time series panels). Their dynamic behaviour at the leading edge of migrating cells was coordinated (lower right kymograph). These results suggest that KLHL21 localizes to dynamic FA structures in migrating cells.

**Figure 2:**
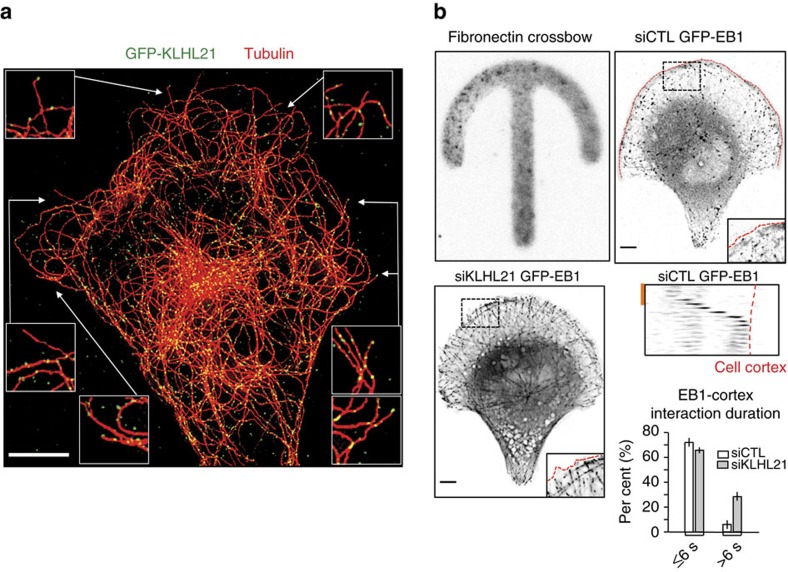
KLHL21 affects EB1–cortex interactions. (**a**) U20S-FRT/TO cells stably expressing GFP-KLHL21 (green) were stained for tubulin (red) and pre-permeabilised to reduce cytoplasmic staining (maximum intensity projection of a 3 image stack in z with 235 nm step size). Scale bar, 5 μm. (**b**) U20S cells expressing GFP-EB1 were plated on fibronectin-coated crossbow micro patterns (Cytoo, upper left panel) and depleted for endogenous EB1. Cells were further treated with either control (siCTRL, upper right) or KLHL21 (siKLHL21, lower left) RNAi-oligos and imaged by wide-field microscopy to visualize EB1 comets (Supplementary Movies 5 and 6). Cell cortex is marked by a red line. Scale bar, 5 μm. The boxed area is magnified in the insets. The lower right panel shows a kymograph (top) and quantitation (bottom). Kymograph is of a single Mt marked with GFP-EB1 reaching the cell cortex (red line, Supplementary Movie 6). Red scale bar, 10 s. The duration (in seconds) of the Mt–cortex interaction and sliding of Mts at the cortex was quantified in HeLa cells expressing GFP-EB1, depleted (siKLHL21) or not (siCTL) for KLHL21 with endogenous EB1 depletion in all conditions. Error bars indicate s.d. between three independent experiments.

In addition, GFP-KLHL21 also accumulated on centrosomes and was found in discrete ‘puncta' at Mt tips (Fig. 2a; Supplementary Fig. 2A) and along Mt (Fig. 2a). Indeed, co-staining with the Mt-plus-end binding protein EB1 showed that GFP-KLHL21 accumulated at Mt tips in over 90% of Mts reaching the cortex in interphase (Supplementary Fig. 2A, over 150 EB1 comets). Accumulation of KLHL21 at Mt-plus ends required EB1, as GFP-KLHL21 staining at Mt tips was strongly diminished in HeLa cells depleted for EB1 but not in cells depleted for Cul3 (Supplementary Fig. 2B). Taken together, we conclude that KLHL21 accumulates at FA structures and interacts with stable Mts and Mt-plus ends in an EB1-dependent manner.

To investigate the molecular mechanism underlying the observed cell migration defect, we analysed Mt network stability in HeLa cells RNAi-depleted for Cul3 or KLHL21 by staining for acetylated tubulin as a measure of Mt stability (Supplementary Fig. 3A). Interestingly, in siCul3- and siKLHL21-treated cells the average Mt acetylation intensity was increased approximately fourfold compared with siRNAi controls, suggesting that the CRL3^KLHL21^ E3 ligase affects microtubule dynamics. To corroborate these results, we quantified EB1 dynamics of individual Mts reaching the cell cortex in U2OS cells stably expressing GFP-EB1 and plated on a defined fibronectin crossbow micro pattern (Cytoo) (Fig. 2b; Supplementary Movie 5). While in RNAi-control cells only few Mts were stabilized at the cortex and most EB1–cortex interactions lasted between 2 and 6 s, many Mt–cortex interactions were extended in cells depleted for KLHL21 (Fig. 2b, *n*=500 per condition, Supplementary Movie 6). Together, these data suggest that CRL3^KLHL21^ affects Mt dynamics and may specifically regulate the turnover of Mts reaching the cell cortex.

### CRL3^KLHL21^ predominantly ubiquitylates lysine 100 of EB1

**Figure 3:**
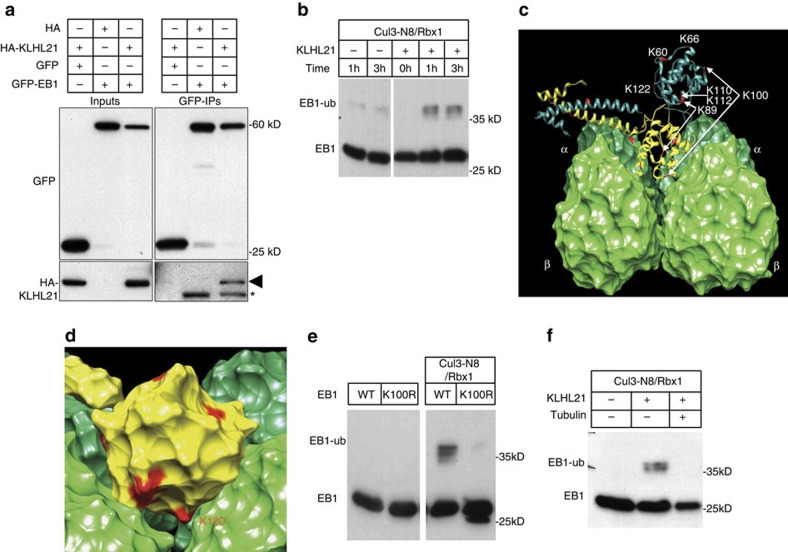
EB1 is preferentially ubiquitylated on lysine 100. (**a**) HeLa cells were co-transfected as indicated with plasmids expressing HA-KLHL21 and GFP-EB1, or the corresponding empty HA- or GFP controls. Cell extracts were incubated with anti-GFP antibodies (GFP-IP) and bound proteins analysed by immunoblotting with anti-GFP (upper panels) or HA antibodies (lower panels). An aliquot of the cell extract was used to control protein expression (input). The black arrow marks HA-KLHL21 co-immunoprecipitating with GFP-EB1, while the asterisk points to GFP-EB1 cross reacting with the secondary antibody used for immunoblotting. (**b**) Purified EB1 was incubated with reconstituted and neddylated (N8) Cul3/Rbx1/UbH5 and ubiquitin (Ub) in the absence (−) or presence (+) of KLHL21 purified from *E. coli*. At the indicated times (hours), the reaction was stopped and analysed by immunoblotting with EB1-antibodies. The position of unmodified (EB1) and ubiquitylated EB1 (EB1-ub) is indicated. (**c**) Schematic representation of a EB1-dimer (yellow and cyan) binding to α-β tubulin (dark and light green, respectively, adapted from Maurer *et al*.^11^ and Slep *et al*.^31^). The positions of the ubiquitylated lysine residues identified by mass spectrometry from the *in vitro* reaction shown in **d** are indicated. (**d**) The position of lysine 100 (K100) was mapped on EB1 surfaces known to be involved in Mt binding. (**e**) Recombinant EB1 or EB1^K100R^ alone (left panel) or with recombinant E2 and E3 enzymes (right panel) were subjected to *in vitro* ubiquitylation reactions as described in **d**. (**f**) Recombinant EB1 was subjected to *in vitro* ubiquitylation reactions with recombinant E2 and E3 enzymes and ubiquitin as described in **d** in the presence (+) or absence (−) of KLHL21 and stabilized Mts. The position of unmodified (EB1) and ubiquitylated EB1 (EB1-ub) is indicated.

To investigate how CRL3^KLHL21^ E3 ligase regulates microtubule dynamics, we analysed the amounts of known Mt-regulators in HeLa cells RNAi depleted for Cul3 and KLHL21 (Supplementary Fig. 3B,C). Immunoblotting revealed that EB1 levels were slightly increased in the absence of Cul3 or KLHL21, in contrast to other Mt-binding proteins MAP4, CLASP2, p150^Glued^ or EB3. Interestingly, KLHL21 also physically interacted with EB1, as shown by immunoprecipitating GFP-EB1 or GFP alone from HeLa cell extracts expressing HA-KLHL21. Indeed, HA-KLHL21 was present in GFP-EB1 precipitates, but absent in GFP or HA controls (Fig. 3a). Moreover, the *Escherichia coli* expressed GST-tagged KELCH domains of KLHL21, but not KLHL22, were able to bind purified EB1 *in vitro* (Supplementary Fig. 4A), although the affinity of this interaction appears very low. Overall, these data indicate that EB1 is able to bind KLHL21, implying that EB1 might be a direct ubiquitylation target of CRL3^KLHL21^. To test this possibility, we performed *in vitro* ubiquitylation reactions using recombinantly produced Cul3-N8/RBX1/KLHL21 (CRL3^KLHL21^) E3 ligase. Interestingly, CRL3^KLHL21^ was able to ubiquitylate a small fraction of EB1 *in vitro* in a KLHL21-dependent manner (Fig. 3b). Mass spectrometry analysis of EB1 ubiquitylation predominantly identified lysine 100 but also lysines K60, K66, K89, K112 and K122 as ubiquitin acceptor sites *in vitro* (Fig. 3c,f, red; Supplementary Table 1), and several of these lysine residues including K100 and K60, have been previously found to be ubiquitylated *in vivo*^30^. Mapping the ubiquitylated lysine residues onto the EB1–Mt structural model (adapted from Maurer *et al*.^11^ and Slep *et al*.^31^) revealed that K89 and K100 are located within the EB1 CH-domain (Fig. 3c,d), and K89 has been previously shown to be crucial for Mt binding^32^.

To identify the predominant lysine residue(s) on EB1 targeted by CRL3^KLHL21^, we purified different non-ubiquitylatable EB1 lysine to arginine mutants (K→R), and subjected them to *in vitro* ubiquitylation reactions. Interestingly, the EB1^K100R^ mutant, in which only lysine K100 was mutated to the non-ubiquitylatable arginine, was a poor substrate *in vitro* compared with wild-type EB1 (Fig. 3e). Conversely, we produced an EB1 mutant in which only lysine K100 and/or K89 were unmodified, but all other lysines residues were mutated to non-ubiquitylatable arginine residues. In contrast to EB1^K100only^ and EB1^K89/K100only^, ubiquitylation of the EB1^K89only^ mutant was strongly reduced (Supplementary Fig. 4B), demonstrating that CRL3^KLHL21^ preferentially ubiquitylates lysine 100 located in the CH-domain of EB1. Importantly, in contrast to EB1^K89R^, EB1^K100R^ displays a microtubule binding affinity that is comparable to wild-type EB1 as determined by microtubule co-pelleting assays *in vitro* (Supplementary Fig. 4D,E), confirming that mutating lysine 100 to an arginine residue preserves the overall Mt-binding activity of EB1. Like K89, K100 is conserved in fission and budding yeast, mouse, xenopus and humans (Supplementary Fig. 4C), and is located between the tubulin heterodimers when the CH-domain of EB1 binds to Mt ends (Fig. 3c,d). Consistent with this analysis, stabilized microtubule addition to the CRL3^KLHL21^ *in vitro* ubiquitylation reaction blocks EB1 ubiquitylation (Fig. 3f), implying that K100 is poorly accessible when bound to Mts and thus EB1 ubiquitylation on K100 is likely to interfere with its ability to bind Mt ends.

### EB1 ubiquitylation may regulate cell migration

**Figure 4:**
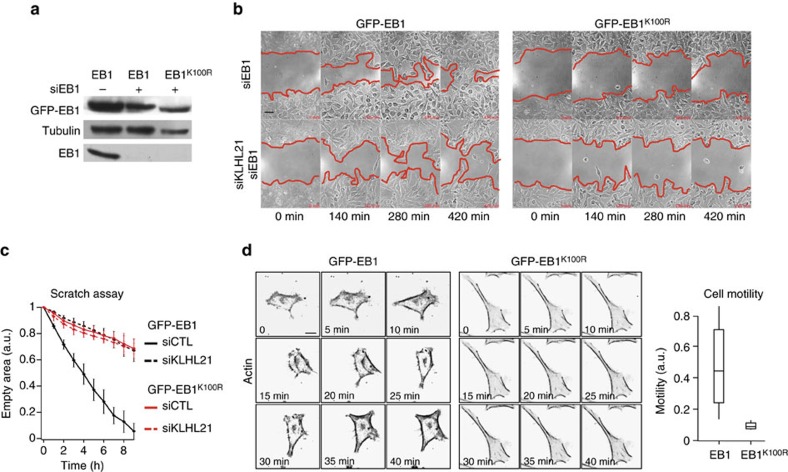
Ubiquitylation of EB1 may regulate cell migration. (**a**) Total cell extracts of HeLa cells stably expressing GFP-EB1 or GFP-EB1^K100R^ from the doxycycline (Dox)-inducible promoter were analysed by immunoblotting. Where indicated (+), cells were treated with siRNA oligos specifically targeting the endogenous copy of EB1. (**b**) Confluent HeLa cultures depleted of endogenous EB1 but stably expressing GFP-EB1 or GFP-EB1^K100R^ were treated with control siRNA (siCTL) or siRNA specifically depleting KLHL21 (siKLHL21) and scratched at time 0. (**c**) The resulting cell-free zone (area within red line) was imaged every hour during closure, and the empty area quantified for each siRNA condition by plotting arbitrary units (a.u.) against the time after scratching (hours). Error bars indicate variability of three independent experiments. (**d**) Images from time lapse movies of individual HeLa cells depleted of endogenous EB1 but expressing either GFP-EB1 or GFP-EB1^K100R^ (Supplementary Movie 3) and stained with SIR-Actin dye. Cell motility was quantified using the Manual Tracking plugin of Image J (scale bar, 20 μm), and expressed as velocity mean (a.u.).

To determine whether CRL3^KLHL21^-dependent EB1 ubiquitylation on lysine 100 may regulate cell migration *in vivo*, we downregulated endogenous EB1 in HeLa cells stably expressing GFP-tagged wild-type or EB1^K100R^ protein from the doxycycline promoter at levels similar to endogenous EB1 (Fig. 4a; Supplementary Fig. 5A). The half-life of EB1^K100R^ was comparable to wild-type controls (Supplementary Fig. 5B), indicating that CRL3^KLHL21^-dependent EB1 ubiquitylation does not affect its stability. Similar to KLHL21 depletion, cells expressing the GFP-EB1^K100R^ mutant were unable to migrate in our scratch assay, in contrast to GFP-EB1 wild-type controls (Fig. 4b,c; Supplementary Fig. 5D). Importantly, depletion of KLHL21 in GFP-EB1^K100R^ cells did not further aggravate this defect, implying that KLHL21 depletion and ubiquitylation of EB1 on K100 are epistatic. The increased EB1 levels in KLHL21-depleted cells are not responsible for the observed migration defect, as depleting EB1 to endogenous levels did not restore cell migration of cells lacking KLHL21 (Supplementary Fig. 5C). Together, these results suggest that CRL3^KLHL21^-dependent ubiquitylation of EB1 on K100 controls cell migration.

To examine whether EB1 ubiquitylation alters motility and cortical morphology, we imaged HeLa cells stably expressing wild-type GFP-EB1 or the GFP-EB1^K100R^ mutant at low magnification and tracked individual cells during 12 h. As GFP-EB1 and GFP-EB1^K100R^ expression levels in stable HeLa cells were heterogeneous within individual clones, we only analysed cells with comparable integrated intensity (maximum ratio of 1 to 2 for GFP-EB1 to GFP-EB1^K100R^). As shown in Fig. 4c, the mobility of individual cells expressing EB1^K100R^ was significantly reduced (Supplementary Movie 7). Moreover, the cortical plasticity of EB1^K100R^-expressing cells was greatly impaired and, in contrast to wild-type EB1 controls, almost no lamellipodia and cortical protrusions were detected (Fig. 4d; Supplementary Fig. 5D,E). As expected, a similar cell motility defect was observed in cells depleted for KLHL21 or CSN2, an essential subunit of the CSN complex (Supplementary Fig. 5E)^22^, confirming that CRL3^KLHL21^ activity is required for this process. Taken together, these data demonstrate that cells expressing a non-ubiquitylable EB1^K100R^ mutant phenotypically mimic KLHL21 depletion with respect to cell migration and cortical dynamics, suggesting that CRL3^KLHL21^ may regulate EB1 at the cell cortex.

### Cortical Mt and FA are altered in EB1^K100R^ mutant

**Figure 5:**
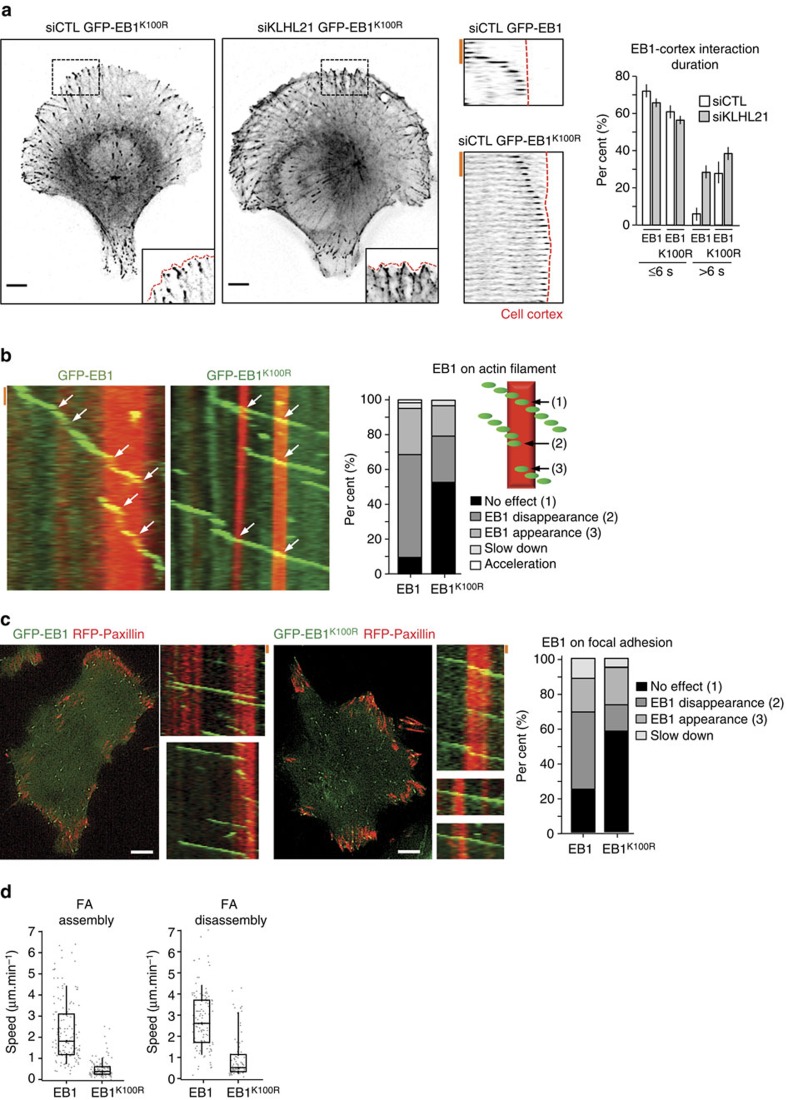
EB1 ubiquitylation on K100 regulates Mt–cortex interactions. (**a**) U2OS cells expressing GFP-EB1 or GFP-EB1^K100R^ were plated on fibronectin-coated crossbow micro patterns (Cytoo) and depleted for endogenous EB1. Cells were treated with either control (siCTRL) or KLHL21 RNAi-oligos (siKLHL21) and imaged by wide field microscopy (Supplementary Movies 5 and 6). Cell images (left panel) show EB1 comets. Cell image insets show the boxed area at higher magnification with cell cortex marked by a red line. Scale bar, 5 μm. Kymographs (middle panels) of a single Mt marked with GFP-EB1 (upper) or GFP-EB1^K100R^ (lower) reaching the cell cortex (red line, Supplementary Movie 4). Red scale bar, 10 s. Graph (right panel) shows duration (seconds) of the Mt–cortex interaction and sliding of Mts at the cortex quantified from HeLa cells expressing GFP-EB1 or GFP-EB1^K100R^, depleted (siKLHL21) or not (siCTL) for KLHL21 with endogenous EB1 depletion in all conditions. Error bars indicate s.d. between three independent experiments. (**b**) U2OS cells depleted for endogenous EB1 and stably expressing GFP-EB1 or GFP-EB1^K100R^ were plated on fibronectin-coated crossbow micro patterns (Cytoo, Supplementary Fig. 6), and stained with the actin dye SIR-actin. Kymographs of individual Mts (indicated by the dotted lines in Supplementary Fig. 6). Individual Mt tips (left image panels) marked by GFP-EB1 or GFP-EB1^K100R^ (green) were analysed as they encounter actin filaments visualized by staining with SIR-dye (red) (Supplementary Movie 8). Kymographs (right panel) were quantified and classified as schematically summarized (drawing) into number of events shown as a per cent (%) where GFP-EB1 is removed from Mt tips (2), is removed but then reappears (3, rescue) or is unaffected and crosses the actin filament (1). Moreover, the per cent (%) of actin filament encounters that slow down or accelerates Mt growth rates were also counted (red scale bar, 4 s). (**c**) Images of HeLa cells expressing GFP-EB1 or GFP-EB1^K100R^ (green) and transfected with RFP-Paxillin plasmid (red) were obtained using RING-TIRF, scale bar, 5 μm. Kymographs (right of image panel) were quantified (right graph panel) and show single cortical MT behaviours when crossing FAs (red scale bar, 10 s). Error bars indicate variability of three independent experiments. (**d**) Focal adhesion speeds were quantified using RING-TIRF microscopy on cells expressing Paxillin-RFP and either wild-type EB1 or the non-ubiquitinylatable EB1^K100R^ mutant. The data are shown as box plots on top of the single measurements (*n*>150 FAs per condition, *P*<1e^−30^ for assembly and *P*<4e^−20^ for disassembly).

To determine the functional consequences of EB1 ubiquitylation, we quantified EB1 dynamics of individual Mts reaching the cell cortex in polarized U2OS cells stably expressing GFP-EB1 or GFP-EB1^K100R^ as described in Fig. 2b. Interestingly, the number of stable GFP-EB1^K100R^-positive microtubules reaching the cell cortex were significantly increased compared with GFP-EB1 controls (Fig. 5a; Supplementary Movie 5 and 6). This effect was not further enhanced by simultaneous depletion of KLHL21, suggesting that CRL3^KLHL21^ may regulate turnover of cortical Mts by ubiquitylating EB1. To corroborate these results, we analysed Mt–actin crosstalk in cortical areas of polarized cells imaged by wide-field microscopy. Cell areas in contact with fibronectin spotted in a crossbow micro pattern (Cytoo) constitute lamellipodia where FA and stress fibres are frequent (Supplementary Fig. 6; Supplementary Movie 8). GFP-EB1 rapidly dissociated from Mt tips in over 60% of the Mt-contacted actin filaments, resulting in slower Mt growth (*n*=250). GFP-EB1^K100R^ mutant protein remained bound in many cases and Mt growth continued (Fig. 5b; Supplementary Movie 8; Supplementary Fig. 6). As KLHL21 accumulates at FA structures, we also analysed the Mt dynamics at FA using RING-TIRF microscopy of HeLa cells expressing RFP-Paxillin and either GFP-EB1 or GFP-EB1^K100R^ (Fig. 5c). Interestingly, this Mt sub-population showed behaviour at FA similar to that observed at actin filaments. While wild-type GFP-EB1 reaching FAs rapidly disassembled, Mts decorated by GFP-EB1^K100R^ were able to cross FAs (insets). We next used RING-TIRF microscopy to compare the stability of FA structures visualized by RFP-paxillin in HeLa cells expressing either wild-type EB1 or the EB1^K100R^ mutant. Strikingly, the assembly and disassembly of FA structures were strongly reduced in cells expressing EB1^K100R^ (Fig. 5d), suggesting that EB1 dynamics and FA-turnover may be coupled. We thus propose that CRL3^KLHL21^-dependent ubiquitylation of EB1 is required for cell migration by regulating crosstalk between Mt–actin and Mt–FA assemblies.

### CRL3^KLHL21^ alters EB1 dynamics at Mt reaching focal adhesions

**Figure 6:**
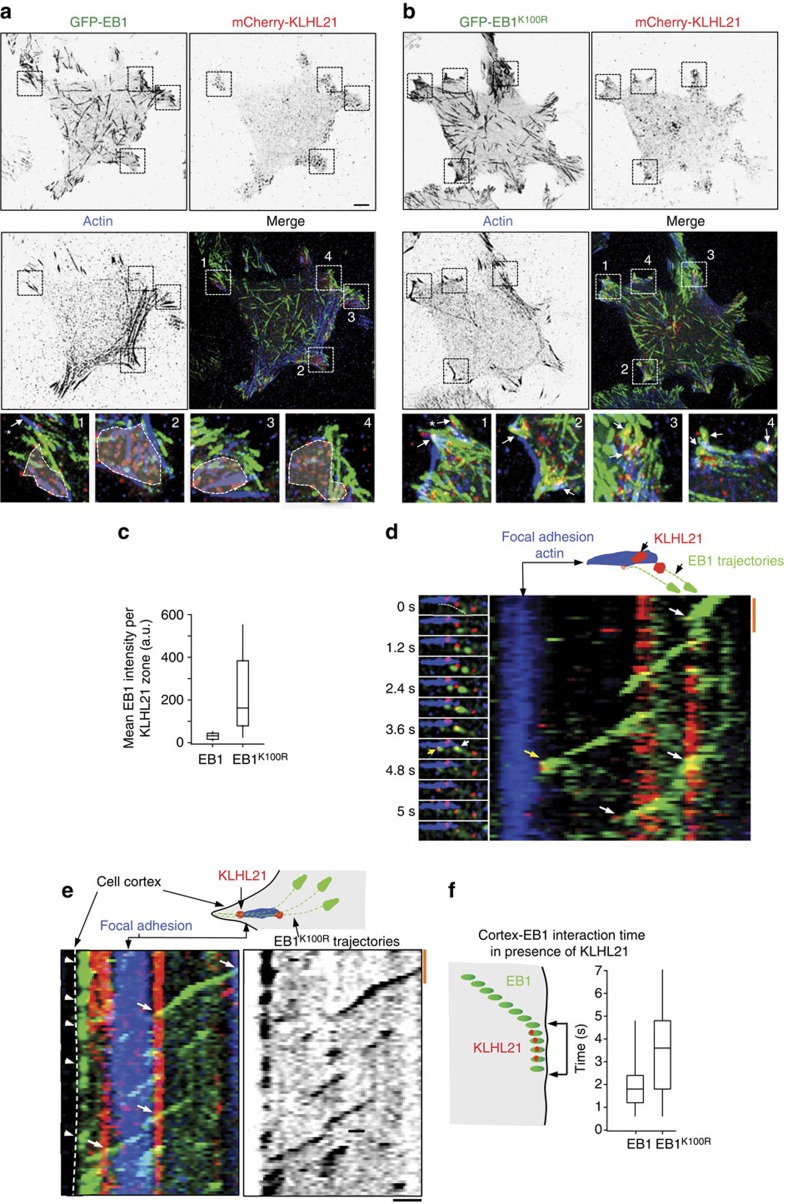
KLHL21 regulates EB1 turnover at FA and actin at cell cortex. HeLa-FRT/TO cells stably expressing GFP-EB1 (**a**) or GFP-EB1^K100R^ (**b**) (green) were transfected with a plasmid expressing RFP-KLHL21 (red) and stained with low doses of SIR-actin dye (blue). The cortex of living cells was imaged using RING-TIRF microscopy, and a time projection is shown (Supplementary Movie 9–11). Individual images depicting GFP-EB1, RFP-KLHL21 and actin as well as merged images are included. Insets (bottom row numbered 1 to 4) show square regions at higher magnification. RFP-KLHL21 patch at FA is circled with a white spotted line. Scale bar, 5 μm. (**c**) Mean EB1 intensity per KLHL21 zone described in **a** and **b** (35 different zones per condition, see Methods). (**d**) Image series (left panel, seconds) and kymograph of GFP-EB1 as in **a** show GFP-EB1 as it reaches the KLHL21-positive FA structure (Supplementary Movie 10). The discontinued line in the corresponding illustration represents the GFP-EB1 trace reaching the FA and recruited RFP-KLHL21 (kymograph, red scale bar: 6 s, horizontal scale bar, 800 nm). The arrows mark EB1-KLHL21 co-localization, preceding disappearance of GFP-EB1 or in some cases pausing of Mt growth. (**e**) Same as **d** with GFP-EB1^K100R^ as in **b** with white arrows to mark GFP-EB1^K100R^ co-localizing with RFP-KLHL21. Red scale bar, 6 s, horizontal scale bar, 1 μm. Red white discontinued line represents cell cortex. (**f**) The duration (in seconds) of individual Mts marked with GFP-EB1 or GFP-EB1^K100R^ co-localizing with RFP-KLHL21 at the cell cortex (bracket) was quantified and shown as a box blot.

To examine the dynamic relationship of KLHL21 and EB1 at FAs, we again used RING-TIRF microscopy to simultaneously image GFP-EB1 or GFP-EB1^K100R^ in live cells transiently expressing mCherry-KLHL21 and stained with SIR-Actin dye. Three-colour nanoscale image sequences were acquired at 600 ms time intervals, and their time projections were analysed (Fig. 6a,b; Supplementary Movie 9–11). Although KLHL21 and EB1 co-localized in multiple Supplementary Movie frames (Supplementary Movie 10), KLHL21 did not behave like a plus-tip-tracking protein. Interestingly, mCherry-KLHL21 clustered around FA, but GFP-EB1 was absent from these clusters (Fig. 6a, enlarged pictures 1–4). In contrast, GFP-EB1^K100R^ readily co-localized with mCherry-KLHL21 in these actin clusters (Fig. 6b, enlarged pictures 1–4). We quantified these data by plotting the mean EB1 intensity per KLHL21 area in cells expressing mCherry-tagged KLHL21 and either wild-type GFP-EB1 or the non-ubiquitylatable GFP-EB1^K100R^ mutant (Fig. 6c). To substantiate this antagonistic behaviour at the cell cortex, we followed individual Mt tips marked by GFP-EB1 as they encounter a FA structure containing KLHL21. Strikingly, GFP-EB1 briefly co-localized with KLHL21, which in turn led to EB1 disappearance (Fig. 6d, white arrows on left panel, Supplementary Movie 9). Indeed, kymographs of GFP-EB1 trajectories confirmed that Mts rapidly pause or shrink after encountering KLHL21 at the cortex (Fig. 6d, left panel, white arrows). In some cases, we also observed *de novo* recruitment of KLHL21 to Mt–FA contact sites, subsequently leading to Mt pause or catastrophe (Fig. 6d, kymograph, yellow arrow, Supplementary Movie 10). In contrast, GFP-EB1^K100R^ comets were not affected when crossing FA and KLHL21 patches (Fig. 6e, white arrows on kymograph, Supplementary Movie 11). Quantification confirmed that the persistence of individual Mt ends marked by GFP-EB1^K100R^ at the cell cortex of KLHL21-containing FA was significantly longer compared with Mt ends marked with wild-type GFP-EB1 (Fig. 6f). We thus conclude that CRL3^KLHL21^ promotes cell motility by ubiquitylating EB1 at the cell cortex and FA structures, thereby triggering its removal from growing Mt ends and affecting FA and cortical dynamics.

## Discussion

Here we show that the CRL3^KLHL21^ E3 ligase controls cell migration at least in part by regulating Mt and FA dynamics during interphase. This novel function adds to our previous finding of CRL3^KLHL21^ activity orchestrating chromosome alignment by regulating Aurora B during mitosis^26^. Indeed, KLHL21 localizes to FA structures at lamellipodia and destabilized FA where in complex with Cul3 it ubiquitylates EB1 at lysine 100 located within the microtubule binding CH-domain. Our data support the notion that CRL3^KLHL21^-dependent mono-ubiquitylation of EB1 dimers inhibits EB1–Mt interactions, thereby promoting microtubule disassembly at actin structures and FAs at the cell cortex of migrating cells.

Dynamic cortical Mts are required for cell migration^2^, possibly by providing directed vesicle transport to lamellipodia. In addition, Mts interact with FAs and are suggested to promote their turnover preferentially at the leading edge^33^,^34^,^35^. Mt growth to reach FA structures does not occur by a random search-and-capture mechanism, but rather is guided along F-actin stress fibres^35^,^36^,^37^. Individual Mts grow multiple times towards the same or different FA, when Mts then frequently pause and switch from growth to shortening^34^. The scaffolding FA component Paxillin regulates these growth-to-shortening transitions^17^, possibly by recruitment or local activation of a factor that terminates Mt growth. Interestingly, CRL3^KLHL21^ fulfils the criteria expected for such a Mt regulator and may alter Mt dynamics by increasing EB1 turnover specifically at cortical actin structures and FAs. Several lines of evidence support the notion that CRL3^KLHL21^ directly ubiquitylates EB1 *in vivo*. First, KLHL21 accumulates at FAs and transiently co-localizes with EB1 bound to tips of Mts reaching FA structures at the leading edge. Second, EB1 co-immunoprecipitates with KLHL21 *in vivo* and binds the KELCH domains of KLHL21 but not the structurally related CRL3-adaptor KLHL22 *in vitro*. Third, purified CRL3^KLHL21^ ubiquitylates EB1 *in vitro* preferentially on lysine 100, which is also found to be ubiquitylated *in vivo*. Finally, cells expressing the non-ubiquitylatable EB1^K100R^ mutant share the striking cell migration and lamellapodia defects observed in KLHL21-depleted cells. We can neither rigorously exclude an EB1-independent function of KLHL21 in cell migration, nor that the EB1^K100R^ mutant affects FA dynamics by a mechanism unlinked to its role in microtubule polymerization. It also remains possible that the lack of CRL3^KLHL21^ activity prevents proper Mt–FA interactions because an unknown disassembly factor is no longer able to reach FA structures under these conditions. Of course, CRL3^KLHL21^ may also target specific FA components in addition to EB1. However, since the cell migration and lamellapodia defects are not further enhanced upon KLHL21 depletion in EB1^K100R^-expressing cells, we conclude that the two proteins are likely to function in the same pathway and that EB1 is the main KLHL21 substrate in this process.

Available evidence suggests that EB1 at growing Mt tips is dynamic with turnover rates of less than one second^7^,^38^. It is therefore unclear why EB1 turnover should be regulated when Mts reach FA structures. Still, EB1 ubiquitylation is likely to directly interfere with its ability to bind Mt tips. Indeed, K100 is located in the EB1 CH-domain, which mediates the interaction with Mt-plus ends by recognizing the GTP cap^11^,^39^,^40^. Modelling lysine 100 on the EB1 bound to α-β tubulin heterodimers predicts a steric clash of ubiquitylated EB1 and Mts. Likewise, Mts addition inhibits CRL3^KLHL21^-mediated EB1 ubiquitylation *in vitro*. Additional steps might be required at the cell cortex to increase the accessibility of the K100 residue and allow its ubiquitylation by CRL3^KLHL21^ *in vivo*. It is conceivable that post-translational modifications of EB1 or other tip-binding proteins, or the mechanical force generated when Mts encounter actin filaments and/or cortical FA structures is sufficient to distort Mt tips. Alternatively, CRL3^KLHL21^ may target-free EB1 dimers to prevent their rebinding and rescue of Mt growth at the cell cortex. It is unlikely that the slightly increased EB1 levels upon CRL3^KLHL21^ depletion explain the cortical Mt-defects, and elevated EB1 levels may rather be an indirect consequence of altered microtubule dynamics. Interestingly, loss of CRL3^KLHL21^ activity also increases tubulin acetylation *in vivo*, which correlates with increased microtubule stability. Increased tubulin acetylation could be caused indirectly by the increased stability of cortical Mts in KLHL21-depleted cells, for example by allowing more time for the acetylation machinery to modify the Mt network. It is also possible that KLHL21 promotes tubulin de-acetylation by an unknown mechanism.

Irrespective of the molecular mechanism controlling cortical Mt dynamics and FA disassembly, it would be interesting to understand how KLHL21 localizes to FA structures preferentially at the leading edge of migrating cells. KLHL21 may be recruited to FA structures by interacting with proteins such as Paxillin, APC, the spectraplakin MACF1/ACF7 or CLASPs, which are all required for directed cell migration. Similar to KLHL21, these proteins localize to FA structures by transiently interacting with EB1 (refs 12, 41). Interestingly, they were implicated in Mt organization and/or stabilization near the leading edge of migrating cells, and CRL3^KLHL21^ could thus stabilize Mt–actin interactions at FA by releasing EB1-associated proteins such as ACF7 or CLASP, which function as Mt–actin crosslinking proteins. Clearly, further work is required to investigate the functional relationship of KLHL21 and these FA factors to understand how they orchestrate Mt and FA dynamics at lamellipodia during cell migration.

## Methods

### Molecular biology and biochemistry

Protein purification is detailed in Supplementary Information. For the *in vitro* ubiquitylation assays (Fig. 3b,e,f; Supplementary Fig. 4B), 0.5 μM Cul3-Nedd8/Rbx1 was mixed with 0.5 μM KLHL21, 0.5 μM EB1 variants and 1 μM E2 (UbcH5b), 0.1 μM ubiquitin E1 (Boston Biochem) and 50 μM ubiquitin (Boston Biochem) in 40 mM Tris/HCl, pH 7.6, 10 mM MgCl_2_, 1 mM ATP and 1 mM DTT, and incubated at 37 °C for 30 min. Reactions were quenched by boiling at 95 °C with LDS-buffer for 5 min and analysed by western blotting.

The Mt co-sedimentation assays were performed as described previously^42^. Purified EB1 was pre-cleared via ultracentrifugation in a TLA-100 rotor for 10 min at 120,000*g* and added at a final concentration of 0.5 μM to variable amounts of taxol-stabilized Mts (Cytoskeleton) in BRB80 buffer (80 mM Pipes pH 6.8, 1 mM MgCl_2_ and 1 mM EGTA). The salt concentration was adjusted in each reaction to 150 mM NaCl final concentration, and incubated for 15 min at room temperature. Subsequently, the supernatant and pellet fractions were separated by ultracentrifugation at 120,000*g* for 20 min at 25 °C, analysed by SDS–PAGE and quantified using ImageJ.

HeLa cell extracts were prepared as described^43^, and HA-tagged KLHL21 immunoprecipitated using anti-HA M2-agarose beads (Sigma). GFP-fused proteins were immunoprecipitated using GFP-Trap agarose beads (Chromotek). Beads were incubated with cell extracts for 2 h at 4 °C under constant rotation. Before elution, beads were washed five times with TBS-T. Tryptic digestion and mass spectrometry analysis was performed as described in detail in the Supplementary Information.

### Cell culture, immunofluorescence and antibodies

HeLa and U2OS cell lines were grown in a humidified incubator at 37 °C and 5% CO_2_ in Dulbecco's modified Eagle medium (Gibco, Invitrogen, Carlsbad, USA) supplemented with 10% fetal calf serum (PAA Laboratories) and 1% penicillin/streptomycin (Gibco). Stable cell lines expressing GFP-EB1, GFP-EB1^K100R^ or GFP- or RFP-tagged KLHL21 were created using HeLa-FRT/TO or U20S FRT/TO host cell lines. Antibiotics for selection were used as follows: hygromycin (Invitrogen) 100 μg ml^−1^, blasticidin (Invitrogen) 15 μg/ml and puromycin (Sigma Aldrich) 0.5 μg ml^−1^. Protein expression was induced by the addition of doxycycline (Sigma) at 0.1 or 1 μg ml^−1^. HeLa cells stably expressing GFP-EB1 or GFP-EB1^K100R^ were transfected with a plasmid expressing RFP-KLHL21 for 24 h.

siRNA transfections were performed using lipofectamine RNAi Max (Invitrogen) at 100 nM final concentration. The following siRNA oligonucleotides (Microsynth) were used: non-silencing control: 5′- UUCUCCGAACGUGUCACGU -3′; Cul3: 5′- AAC AAC UUU CUU CAA ACG CUA -3′); CSN2: 5′- AAG CGG CAU UAA GCA GUU UCC -3′; EB1: Abnova (ref: H00022919-R01), siRNA against KLHL21 and KLHL22 have been described previously^26^,^27^. siRNA transfection were performed for at least 48 h and up to 72 h.

To measure cell mobility by scratch assay, HeLa stably expressing GFP-EB1 or GFP-EB1^K100R^ were grown to confluency in 6-well plates, treated with siRNA (day 0) and imaged directly using a dry 20 × magnification objective. Scratches were performed with a tip burned Pasteur pipette. To measure cell motility of single cells, they were plated at low density (day 1) and imaged (day 1 and 2), quantified using the Manual tracker plugin from ImageJ and plotted using Igor software (Wavemetrics).

For immunofluorescence analysis, cells were grown on high precision cover slips (Zeiss), fixed with −20 °C methanol for ∼8 min, blocked for 30 min in PBS+FCS (5%), incubated for 1 h with primary antibody and then washed three times with PBS+0.05% Tween. Bound antibodies were detected by fluorescently labelled secondary antibodies that were incubated for one hour, washed three times with PBS+0.05% Tween, and mounted with Vectashield H-1000. Cell lines expressing GFP-tagged proteins were treated with GFP booster (Chromotek) as a secondary antibody (1:300). Actin was stained over night with SIR-actin dye (Spirochrome) following instructions by the manufacturer.

The following antibodies (with supplier and used dilution) were used in this study: mouse acetylated tubulin (Abcam ab24610, 1:1,000), mouse EB1 (BD biosciences 610534, 1:5,000), rabbit alpha tubulin (Abcam ab11270, 1:10,000), rabbit KLHL22 (ref. 27), rabbit KLHL21 (ref. 26; 1:200), mouse Aurora B (BD Biosciences 61108/3, 1:1,000), rat Clasp2 (Absea 032006E03, 1:2,000), mouse EB3 (BD bioscience 612156, 1:1,000), mouse p150 glued (BD science 610474, 1:2,000), mouse GFP (Roche 11 814460 001, 1:10,000), rabbit Paxillin (Abcam ab32084, 1:1,000), rabbit VASP (Protein Tech 13472-1-AP, 1:1,000), and rabbit MAP4 (Abcam ab89650, 1:500).

Full blots of Fig. 3b,e,f are provided in Supplementary Fig. 7.

### Microscopy

For live cell microscopy, cells were imaged using L-15 Lebovitz media with 10% FCS in labtek chambers, or plated on large crossbow micro patterns from Cytoo (ref. 10-006-10-18). Images were acquired on fully automated inverted epi-fluorescence microscopes (Ti-Eclipse, Nikon) in an incubation chamber set to 37 °C, with 100 × oil objectives and appropriate excitation and emission filters. A motorized XY-stage and piezo drive was used to acquire z-stacks and multiple fields of view per time point. Cells grown on micro patterns were analysed in a modified Cytoo chamber (ref. 30-011) that fits into a homemade high precision multi holder for the Nikon Ti-Eclipse microscope.

All super resolution images were acquired on a OMX V4 at a laser intensity as low as possible (<33%) and exposure times between 5 and 200 ms. Images acquired with the TIRF unit were taken in sequential mode to avoid wavelength crosstalk. Exposure time and laser intensity were set to minimize photobleaching and phototoxicity.

### Image analysis

Motility quantification is performed by measuring empty area at each time point and divided by the initial empty area (a.u.). Fifteen different scratches per condition have been performed and those experiments have been reproduced three times. Error bars in the graphic's represent standard deviation between experiments (Figs 1a and 3c; Supplementary Fig. 5C).

To quantify EB1–cortex interactions, U2OS cells stably expressing GFP-EB1 or GFP-EB1^K100R^ were siRNA-treated in 6-well plates (day 0) and incubated with doxycycline (1 μg ml^−1^) for 24 h. Cells were trypsinized, plated on fibronectin-coated crossbow micro patterns (Cytoo) and then imaged every 500 ms (three z-stack of 0.435 μm step, Supplementary Movie 2, 3 and 9). Cells were carefully compared for their EB1 or EB1^K100R^ expression levels, and cells with integrated intensity higher than twofold than the mean integrated intensity were excluded. Stack images were deconvolved using Huygens remote software with the same settings for all conditions. EB1 retention time at the cell cortex was quantified using a line scan on the front edge, and analysed by the Image J ‘Multiple kymograph' plugin with 5 pixels line width. Vertical lines were counted as Mt–cortex interaction and the line size in pixel shows the duration of the interaction. Oblique lines were counted as sliding Mts. 500 events in at least 10 cells were analysed per condition. A *t*-test on the mean was performed using IGOR software (Wavemetrics).

Single cell motility experiment (Fig. 4d; Supplementary Fig. 5D,E) was performed using Image J Manual Tracker plugin and velocity was corrected for any microscope shift. Each cell velocity was averaged per movie. One hundred and fifty cells per condition were collected.

Mean EB1 intensity per KLHL21 zone (Fig. 6c) were quantified as followed; time projections of KLHL21-RFP (red), SIR-Actin (blue) and GFP-EB1 were collected, the KLHL21 patch zones defined as shown in Fig. 6a and the EB1 mean intensity in this zone was then measured. A control zone (no KLHL21 and no EB1 signal) was also determined and EB1 mean intensity in the control zone was deducted from the EB1 mean intensity in the KLHL21 zone. Thirty-five patch zones per condition were collected and presented as box plot. A *t*-test on the mean was performed using IGOR software (Wavemetrics).

Focal adhesion dynamics (Fig. 5d) was measured in HeLa cell stably expressing either GFP-EB1 or GFP-EB1^K100R^ and transfected a plasmid encoding Paxillin-RFP. Cell were imaged using RING-TIRF microscopy, and the speed of FA assembly and disassembly was calculated using kymographs. More than 20 cells per condition and at least 10 dynamic FA's per cell were collected (*n*>150 FA per condition). Box plot and *t*-test on the mean have been performed using IGOR pro software (Wavemetrics).

Acetylated tubulin was determined using immunofluorescent staining with specific antibodies. Signal intensity was quantified for at least 30 cells per condition in three independent experiments and plotted as the average per cell with standard deviation calculated from the mean.

For all experiments, cells were chosen blindly, without any exclusion criteria. Every experiment was reproduced at least three times, except for Fig. 5d, where cells were collected from two independent experiments and mixed to obtain a single cell pool per condition. All *t*-test on the mean data (Figs 4d and 5f) meet the stringent assumptions of the test.

### Stable cell lines

Standard protocols were used for DNA and RNA manipulations. EB1 mutants were generated with Pfu Turbo DNA polymerase (Stratagene), using the primers 5′- TTAGTAAAAGGAAGGTTTCAGGACAAT -3′ and 5′- ATTGTCCTGAAACCTTCCTTTTACTAA -3′, and verified by sequencing.

The DNA sequence was introduced in a pcdna5 vector and transfected in HeLa or U2OS FRT/TO host cell lines. Clone selection was performed first by Western blot, and clones expressing more than two times compared with endogenous protein were rejected. Selected clones were then screened with a fluorescent microscope to exclude clones showing high heterogeneity in GFP (or RFP)-tagged protein levels.

### Data availability

The data that support the findings of this study are available from the corresponding author upon request.

## Additional information

**How to cite this article:** Courtheoux, T. *et al*. Cortical dynamics during cell motility are regulated by CRL3^KLHL21^ E3 ubiquitin ligase. *Nat. Commun.* 7:12810 doi: 10.1038/ncomms12810 (2016).

## Supplementary Material

Supplementary Information Supplementary Figures 1-7, Supplementary Table 1, Supplementary Methods,

Supplementary Movie 1 Confluent HeLa cells treated with the indicated siRNA oligos after scratching at time 0.

Supplementary Movie 2 HeLa cells stably expressing GFP-KLHL21 and stained with SIR-Actin dye. The cell cortex was imaged by RING TIRF microscopy.

Supplementary Movie 3 HeLa cells stably expressing GFP-KLHL21 and transiently expressing RFP-Paxillin. The cell cortex was imaged by RING TIRF microscopy

Supplementary Movie 4 GFP-KLHL21 and Paxillin at cell cortex-Mobile cell. HeLa cells stably expressing GFP-KLHL21 and transiently expressing RFP-Paxillin. The cell cortex was imaged by RING TIRF microscopy during the cell cycle.

Supplementary Movie 5 Mt dynamic on crossbow micro-pattern. U2OS cells siRNA-depleted for endogenous EB1 but expressing either GFP-EB1 or GFP-EB1K100R were plated on fibronectin-coated crossbow micro patterns (Cytoo{copyright, serif}). The cells were treated with either control (siCTRL) or siRNA-oligos targeting KLHL21 (siKLHL21) and imaged by wide field microscopy.

Supplementary Movie 6 Cortex enlarged-Mt dynamic on crossbow micro-pattern. Enlarged zone from movie 5.

Supplementary Movie 7 Single cell motility. HeLa cells stably expressing GFP-EB1 or GFP-EB1K100R and treated with siRNA as indicated. Cortical dynamics were imaged for several hours.

Supplementary Movie 8 GFP-EB1 or GFP-EB1K100R and actin dynamics. U2OS cells expressing GFP-EB1 or GFP-EB1K100R (green) were plated on fibronectin-coated crossbow micro patterns (Cytoo{copyright, serif}) and stained with SIR-Actin dye (red).

Supplementary Movie 9 GFP-EB1, RFP-KLHL21 and actin dynamics at cell cortex. HeLa cells stably expressing GFP-EB1 (green) and transiently expressing RFP-KLHL21 (red) were stained with SIR-Actin dye (blue). The cell cortex was imaged by RING TIRF microscopy.

Supplementary Movie 10 Enlarged zone from movie 9. Inset of an EB1 comet reaching a KLHL21 spot at an actin fibber from movie 9.

Supplementary Movie 11 GFP-EB1, RFP-KLHL21 and actin dynamics at cell cortex. HeLa cells stably expressing GFP-EB1K100R (green) and transiently expressing RFP-KLHL21 (red) were stained with SIR-Actin dye (blue). The cell cortex was imaged by RING TIRF microscopy.

## References

[b1] Akhshi T. K., Wernike D. & Piekny A. Microtubules and actin crosstalk in cell migration and division. Cytoskeleton (Hoboken) 71, 1–23 (2014). 24127246 10.1002/cm.21150

[b2] Vasiliev J. M. . Effect of colcemid on the locomotory behaviour of fibroblasts. J. Embryol. Exp. Morphol. 24, 625–640 (1970). 4923996

[b3] Mitchison T. & Kirschner M. Dynamic instability of microtubule growth. Nature 312, 237–242 (1984). 6504138 10.1038/312237a0

[b4] Wloga D. & Gaertig J. Post-translational modifications of microtubules. J. Cell Sci. 123, 3447–3455 (2010). 20930140 10.1242/jcs.063727 PMC2951466

[b5] Jiang K. & Akhmanova A. S. Microtubule tip-interacting proteins: a view from both ends. Curr. Opin. Cell Biol. 23, 94–101 (2011). 20817499 10.1016/j.ceb.2010.08.008

[b6] Sandblad L. . The Schizosaccharomyces pombe EB1 homolog Mal3p binds and stabilizes the microtubule lattice seam. Cell 127, 1415–1424 (2006). 17190604 10.1016/j.cell.2006.11.025

[b7] Bieling P. . Reconstitution of a microtubule plus-end tracking system *in vitro*. Nature 450, 1100–1105 (2007). 18059460 10.1038/nature06386

[b8] Bieling P. . CLIP-170 tracks growing microtubule ends by dynamically recognizing composite EB1/tubulin-binding sites. J. Cell Biol. 183, 1223–1233 (2008). 19103809 10.1083/jcb.200809190 PMC2606963

[b9] Vitre B. . EB1 regulates microtubule dynamics and tubulin sheet closure *in vitro*. Nat. Cell Biol. 10, 415–421 (2008). 18364701 10.1038/ncb1703

[b10] Dixit R. . Microtubule plus-end tracking by CLIP-170 requires EB1. Proc. Natl Acad. Sci. USA 106, 492–497 (2009). 19126680 10.1073/pnas.0807614106 PMC2626730

[b11] Maurer S. P., Fourniol F. J., Bohner G., Moores C. A. & Surrey T. EBs recognize a nucleotide-dependent structural cap at growing microtubule ends. Cell 149, 371–382 (2012). 22500803 10.1016/j.cell.2012.02.049 PMC3368265

[b12] Akhmanova A. S. & Steinmetz M. O. Tracking the ends: a dynamic protein network controls the fate of microtubule tips. Nat. Rev. Mol. Cell Biol. 9, 309–322 (2008). 18322465 10.1038/nrm2369

[b13] Honnappa S. . An EB1-binding motif acts as a microtubule tip localization signal. Cell 138, 366–376 (2009). 19632184 10.1016/j.cell.2009.04.065

[b14] Jiang K. . A proteome-wide screen for mammalian SxIP motif-containing microtubule plus-end tracking proteins. Curr. Biol. 22, 1800–1807 (2012). 22885064 10.1016/j.cub.2012.07.047

[b15] Wolfenson H., Henis Y. I., Geiger B. & Bershadsky A. D. The heel and toe of the cell's foot: a multifaceted approach for understanding the structure and dynamics of focal adhesions. Cell Motil. Cytoskeleton 66, 1017–1029 (2009). 19598236 10.1002/cm.20410 PMC2938044

[b16] Stehbens S. & Wittmann T. Targeting and transport: how microtubules control focal adhesion dynamics. J. Cell Biol. 198, 481–489 (2012). 22908306 10.1083/jcb.201206050 PMC3514042

[b17] Efimov A. . Paxillin-dependent stimulation of microtubule catastrophes at focal adhesion sites. J. Cell Sci. 121, 196–204 (2008). 18187451 10.1242/jcs.012666 PMC3164837

[b18] Palazzo A. F., Eng C. H., Schlaepfer D. D., Marcantonio E. E. & Gundersen G. G. Localized stabilization of microtubules by integrin- and FAK-facilitated Rho signaling. Science 303, 836–839 (2004). 14764879 10.1126/science.1091325

[b19] Hershko A. The ubiquitin system for protein degradation and some of its roles in the control of the cell division cycle. Cell Death Differ. 12, 1191–1197 (2005). 16094395 10.1038/sj.cdd.4401702

[b20] Li W. & Ye Y. Polyubiquitin chains: functions, structures, and mechanisms. Cell Mol. Life Sci. 65, 2397–2406 (2008). 18438605 10.1007/s00018-008-8090-6 PMC2700825

[b21] Lydeard J. R., Schulman B. A. & Harper J. W. Building and remodelling Cullin-RING E3 ubiquitin ligases. EMBO Rep. 14, 1050–1061 (2013). 24232186 10.1038/embor.2013.173 PMC3849489

[b22] Enchev R. I., Schulman B. A. & Peter M. Protein neddylation: beyond cullin-RING ligases. Nat. Rev. Mol. Cell Biol. 16, 30–44 (2015). 25531226 10.1038/nrm3919 PMC5131867

[b23] Kobayashi A. . Oxidative stress sensor Keap1 functions as an adaptor for Cul3-based E3 ligase to regulate proteasomal degradation of Nrf2. Mol. Cell Biol. 24, 7130–7139 (2004). 15282312 10.1128/MCB.24.16.7130-7139.2004 PMC479737

[b24] Cullinan S. B., Gordan J. D., Jin J., Harper J. W. & Diehl J. A. The Keap1-BTB protein is an adaptor that bridges Nrf2 to a Cul3-based E3 ligase: oxidative stress sensing by a Cul3-Keap1 ligase. Mol. Cell Biol. 24, 8477–8486 (2004). 15367669 10.1128/MCB.24.19.8477-8486.2004 PMC516753

[b25] Jin L. . Ubiquitin-dependent regulation of COPII coat size and function. Nature 482, 495–500 (2012). 22358839 10.1038/nature10822 PMC3292188

[b26] Maerki S. . The Cul3-KLHL21 E3 ubiquitin ligase targets aurora B to midzone microtubules in anaphase and is required for cytokinesis. J. Cell Biol. 187, 791–800 (2009). 19995937 10.1083/jcb.200906117 PMC2806313

[b27] Beck J. . Ubiquitylation-dependent localization of PLK1 in mitosis. Nat. Cell Biol. 15, 430–439 (2013). 23455478 10.1038/ncb2695 PMC7116173

[b28] Gao D. . Organoid cultures derived from patients with advanced prostate cancer. Cell 159, 176–187 (2014). 25201530 10.1016/j.cell.2014.08.016 PMC4237931

[b29] Ellefsen K. L., Dynes J. L. & Parker I. Spinning-spot shadowless TIRF microscopy. PLoS ONE 10, e0136055 (2015). 26308212 10.1371/journal.pone.0136055 PMC4550233

[b30] Wagner S. A. . A proteome-wide, quantitative survey of *in vivo* ubiquitylation sites reveals widespread regulatory roles. Mol. Cell Proteomics 10, M111.013284–M111.013284 (2011). 10.1074/mcp.M111.013284 PMC3205876 21890473

[b31] Slep K. C. . Structural determinants for EB1-mediated recruitment of APC and spectraplakins to the microtubule plus end. J. Cell Biol. 168, 587–598 (2005). 15699215 10.1083/jcb.200410114 PMC2171753

[b32] Hayashi I. Crystal structure of the amino-terminal microtubule-binding domain of end-binding protein 1 (EB1). J. Biol. Chem. 278, 36430–36434 (2003). 12857735 10.1074/jbc.M305773200

[b33] Rinnerthaler G., Geiger B. & Small J. V. Contact formation during fibroblast locomotion: involvement of membrane ruffles and microtubules. J. Cell Biol. 106, 747–760 (1988). 3126193 10.1083/jcb.106.3.747 PMC2115107

[b34] Kaverina I., Rottner K. & Small J. V. Targeting, capture, and stabilization of microtubules at early focal adhesions. J. Cell Biol. 142, 181–190 (1998). 9660872 10.1083/jcb.142.1.181 PMC2133026

[b35] Krylyshkina O. . Nanometer targeting of microtubules to focal adhesions. J. Cell Biol. 161, 853–859 (2003). 12782685 10.1083/jcb.200301102 PMC2172972

[b36] Kodama A., Karakesisoglou I., Wong E., Vaezi A. & Fuchs E. ACF7: an essential integrator of microtubule dynamics. Cell 115, 343–354 (2003). 14636561 10.1016/s0092-8674(03)00813-4

[b37] Small J. V. & Kaverina I. Microtubules meet substrate adhesions to arrange cell polarity. Curr. Opin. Cell Biol. 15, 40–47 (2003). 12517702 10.1016/s0955-0674(02)00008-x

[b38] Dragestein K. A. . Dynamic behavior of GFP-CLIP-170 reveals fast protein turnover on microtubule plus ends. J. Cell Biol. 180, 729–737 (2008). 18283108 10.1083/jcb.200707203 PMC2265578

[b39] Zanic M., Stear J. H., Hyman A. A. & Howard J. EB1 recognizes the nucleotide state of tubulin in the microtubule lattice. PLoS ONE 4, e7585 (2009). 19851462 10.1371/journal.pone.0007585 PMC2761489

[b40] Maurer S. P., Bieling, Cope J., Hoenger A. & Surrey T. GTPγS microtubules mimic the growing microtubule end structure recognized by end-binding proteins (EBs). Proc. Natl Acad. Sci. USA 108, 3988 (2011). 21368119 10.1073/pnas.1014758108 PMC3053978

[b41] Kumar P. & Wittmann T. +TIPs: SxIPping along microtubule ends. Trends Cell Biol. 22, 418–428 (2012). 22748381 10.1016/j.tcb.2012.05.005 PMC3408562

[b42] Lampert F., Hornung P. & Westermann S. The Dam1 complex confers microtubule plus end-tracking activity to the Ndc80 kinetochore complex. J. Cell Biol. 189, 641–649 (2010). 20479465 10.1083/jcb.200912021 PMC2872915

[b43] Sumara I. . A Cul3-based E3 ligase removes Aurora B from mitotic chromosomes, regulating mitotic progression and completion of cytokinesis in human cells. Dev. Cell 12, 887–900 (2007). 17543862 10.1016/j.devcel.2007.03.019

