# Corrigendum: Cortical dynamics during cell motility are regulated by CRL3^KLHL21^ E3 ubiquitin ligase

**DOI:** 10.1038/ncomms13487

**Published:** 2016-10-31

**Authors:** Thibault Courtheoux, Radoslav I. Enchev, Fabienne Lampert, Juan Gerez, Jochen Beck, Paola Picotti, Izabela Sumara, Matthias Peter

Nature Communications **7**: Article number: 1281010.1038/ncomms12810 (2016); Published: 09 19 2016; Updated: 10 31 2016

References cited within the Supplementary Methods section of this Article were not identified in the Supplementary References section. The references are as follows:

44. Enchev, R. I., Schreiber, A., Beuron, F. & Morris, E. P. Structural insights into the COP9 signalosome and its common architecture with the 26S proteasome lid and eIF3. *Structure* **18,** 518–527 (2010).

45. Enchev, R. I. *et al.* Structural basis for a reciprocal regulation between SCF and CSN. *Cell Rep.* **2,** 616–627 (2012).

46. Elias, J. E. & Gygi, S. P. Target-decoy search strategy for increased confidence in large-scale protein identifications by mass spectrometry. *Nat. Methods* **4,** 207–214 (2007).

