# Supplementary material for: Cortical dynamics during cell motility are regulated by CRL3KLHL21 E3 ubiquitin ligase
Source: Nat Commun. 2016 Sep 19;7:12810. doi: 10.1038/ncomms12810 (PMC5031805; doi:10.1038/ncomms12810)
Supplement: Supplementary Information — Supplementary Figures 1-7, Supplementary Table 1, Supplementary Methods, [file ncomms12810-s1.pdf]

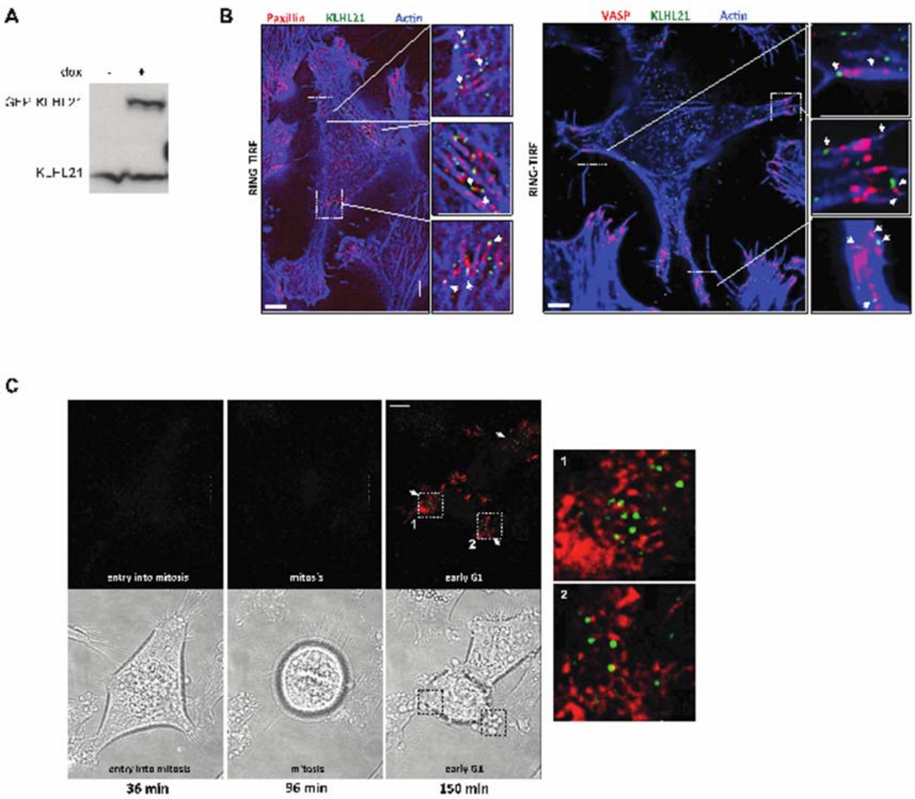

Supplementary Figure 1:
(**A**) HeLa cells stably expressing GFP-KLHL21 under doxycycline promoter were collected and immuno-blotted for KLHL21. Expression was induced (**+**) or not (-) by the addition of doxycycline (dox). (**B**) HeLa cell stably expressing GFP-KLHL21 (green) and stained for Actin with SIR-Actin (blue) and Paxillin or VASP (red). White arrows show KLHL21 localization at FA. Scale bar: 5 µm. (**C**) HeLa-FRT/TO cells from Fig. 1C stably expressing GFP-KLHL21 (green) and transfected with RFP-Paxillin (red) plasmid to mark FAs were observed using RING-TIRF for several hours. Insets show boxed regions at higher magnification. Scale bar: 5 µm. Note that FA adhesion structures are known to disassemble upon entry into mitosis, and are only re-established after cytokinesis in early G1-phase.

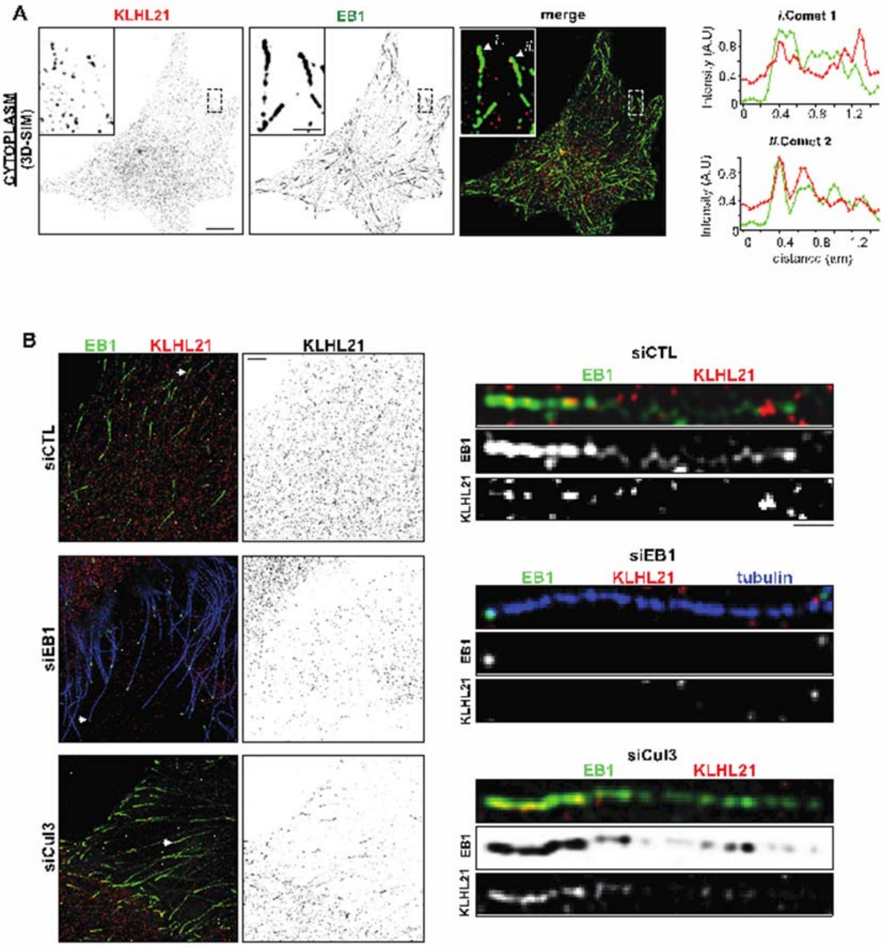

Supplementary Figure 2: (A) Immuno fluorescence analysis of HeLa cells expressing endogenous EB1 (middle) and GFP-KLHL21 (left) using 3D-SIM super-resolution microscopy and shown as merged images (right). Scale bar: 5 mm. A region of interest (square dashed line) is shown at higher magnification in the insets (upper left corner, maximum intensity projection of a 3 image stack in z with 235nm step size at cell cortex proximity). Scale bar: 1 µm. Quantification (right panel) of GFP-KLHL21 (red curve) and EB1 (green curve) signal intensities at the enlarged Mt plus ends (white arrowhead, inset) plotted against the distance (mm) from the Mt tip marked by EB1 staining. **(B)** Localization of endogenous EB1 (left panel) and GFP-KLHL21 (left and right panel) was analysed by 3D-SIM super-resolution microscopy (SRM) in HeLa cells treated with RNAi-controls (siCTL, right upper panels) or RNAi-oligos down-regulating EB1 (siEB1, right middle panels) or Cul3 (siCul3, right lower panels). Scale bar left panels: 5 µm. Individual Mt tips marked with white arrows in the left panel are shown magnified in right panels. EB1 (middle panel), KLHL21 (lower panels) and merged images (top panels) are shown. Scale bar right panels: 0.6 µm.

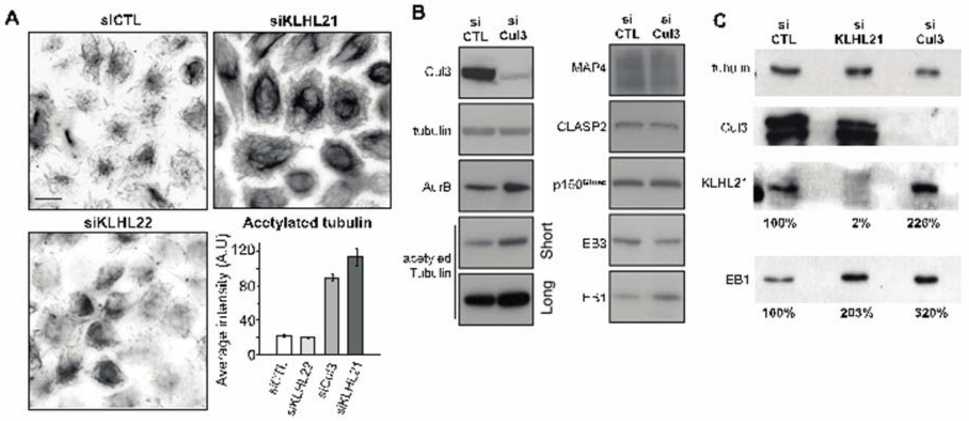

Supplementary Figure 3:
(A) Acetylated tubulin was analysed by immunofluorescence in HeLa cells treated with the indicated siRNA oligos for 48h. Image acquisition settings and treatments were the same for all conditions. Scale bar: 5 μm. The average intensity of acetylated tubulin per cell (image panels) was quantified (lower right graph panel). **(B)** and **(C)** Total cell extracts prepared from HeLa cells treated for 48 hours with RNAi control (siCTL) or with RNAi targeting Cul3 (siCul3) or KLHL21 (siKLHL21) were analysed by immunoblotting with antibodies against the indicated proteins. Tubulin was used to normalize for equal loading. Note that total KLHL21, Aurora B and EB1 levels are slightly increase in the absence Cul3. Quantifications in panel C were performed using the blot analyser from Image J., and expressed as percent (%) protein levels compared to siCTL controls.

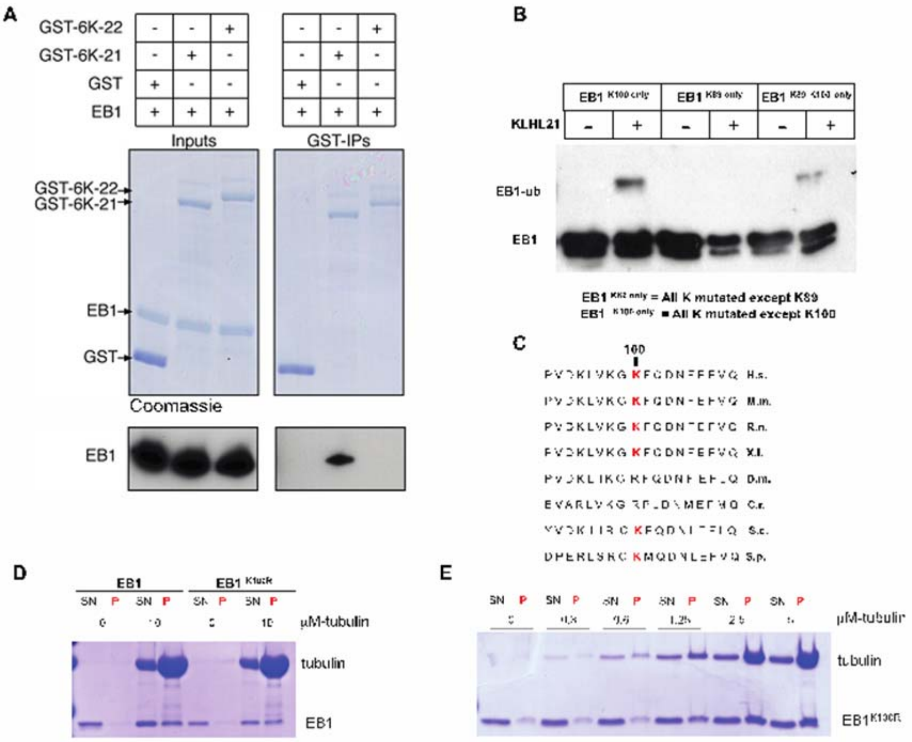

Supplementary Figure 4:
(**A**) Recombinant GST or GST fused to 6 Kelch domains of KLHL21 (GST-6K-K21) or for control KLHL22 (GST-6K-K22) was incubated with recombinant EB1, captured on glutathione–sepharose beads (GST-IP) and bound proteins analysed by Coomassie blue staining (upper panels) and western blotting against EB1 (lower panels). An aliquot of the binding assay was used to control protein expression (input). (**B**) The indicated EB1 mutants were purified and incubated with reconstituted and neddylated Cul3-Nedd8/ Rbx1 complexes and UbcH5 E2 enzyme, with (+) or without (-) KLHL21 expressed in *E.coli*. Lysine 60, 66, 112, 113, 122, 148, 150, 151 182, and 220 of EB1 were mutated to arginine residues (K to R), and thus only K89 and K100 remain available for ubiquitylation. EB1-K100 has in addition K89 changed to arginine, while EB1-K89 has also K100 mutated. Note that EB1 is preferentially ubiquitylated on K100. (**C**) Alignment of the amino acid sequences encompassing K100 (bold) of EB1 from different organisms. Note that K100 is conserved in most species, with a notable exception of *Drosophila*. H.s: Homo sapiens, M.m: *Mus musculus* ,R.n: *Rattus norvegicus*, X.l: *Xenopus laevis*, D.m: *Drosophilia melanogaster*, C.r: *Chlamydomonas reinhardtii*, S.c: *S.cerevisae*, S.p: *S.pombe*. (**D**) and (**E**) Mt pelleting assay using purified wild-type EB1 or the EB1^{K100R} mutant. EB1 was incubated with the indicated concentration of tubulin, Mt polymers separated by centrifugation and the supernatant (SN) and pellet (P) fractions were analysed by SDS-PAGE and Commassie staining.

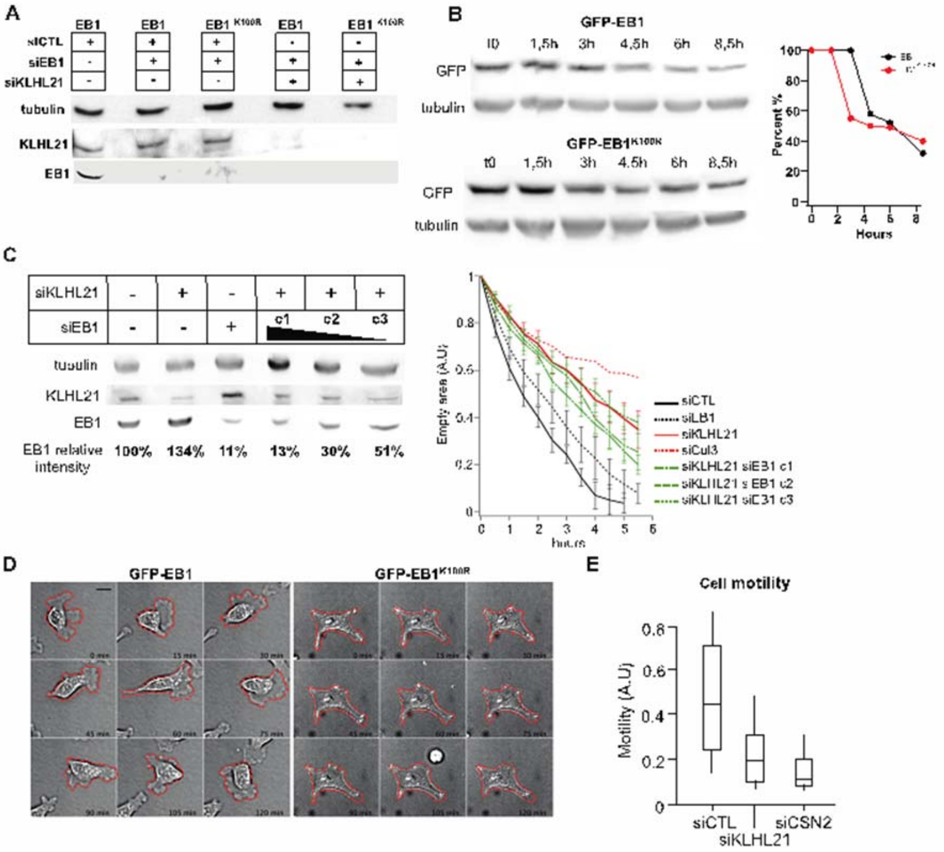

Supplementary Figure 5: Actin and microtubule dynamics in cells expressing GFP-EB1 or GFP-EB1^{K100R}.

(**A**) Western blot of endogenous tubulin, EB1 and KLHL21 of cell extracts prepared from HeLa expressing either GFP-EB1 or GFP-EB1^{K100R} treated as indicated with either control RNAi-oligos (siCTL) or RNAi-oligos targeting KLHL21 (siKLHL21) or endogenous but not exogenously expressed EB1 (siEB1). (**B**) HeLa cell stably expressing GFP-EB1 or GFP-EB1^{K100R} were induced for 24 hours with doxycycline (1mg/ml) and treated with Cycloheximide (15mg/ml). Cells were collected, harvested every 1.5 hours and western blotted. EB1 signal was quantified using Image J tool and normalized to tublin signal for each time point. Values are ploted on the right graph. (**C**) HeLa cells were treated with siRNA KLHL21 and different EB1 siRNA concentrations for 24 hours. EB1 levels are normalized to tubulin signal and values are reported below western blot. Quantification (right graph panel) of scratch assay under conditions described for the left panel. (**D**) Time lapse movies of individual HeLa cells depleted of endogenous EB1 but expressing either GFP-EB1 or GFP-EB1^{K100R} (Scale bar 5 μm, movie 7). The discontinued red lines highlight the cell cortex. (**E**) The cell motility of HeLa cells RNAi-treated with siControl (siCTL) or si-oligos targeting as indicated KLHL21 or CSN2 was quantified using the Manual Tracking plugin of Image J (scale bar: 20 μm), and expressed as arbitrary units (A.U).

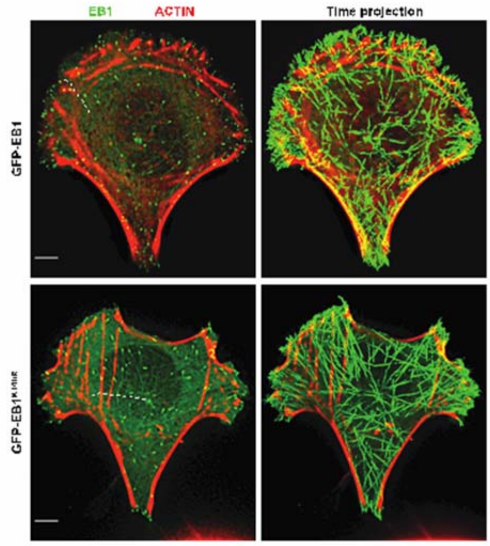

Supplementary Figure 6:
U20S cells from Fig. 4B depleted for endogenous EB1 but stably expressing GFP-EB1 or GFP-EB1^{K100R} were plated on fibronectin-coated crossbow micro patterns (Cytoo©), and stained with the actin dye SIR-actin. GFP-EB1 (upper panels) or GFP-EB1^{K100R} (lower panels) were then visualized together with actin by super-resolution microscopy and shown as a single image (left) or as a time projection (right). White discontinued lines show Mt trajectories from kymographs in Fig. 4B. Scale bar: 5 μm.

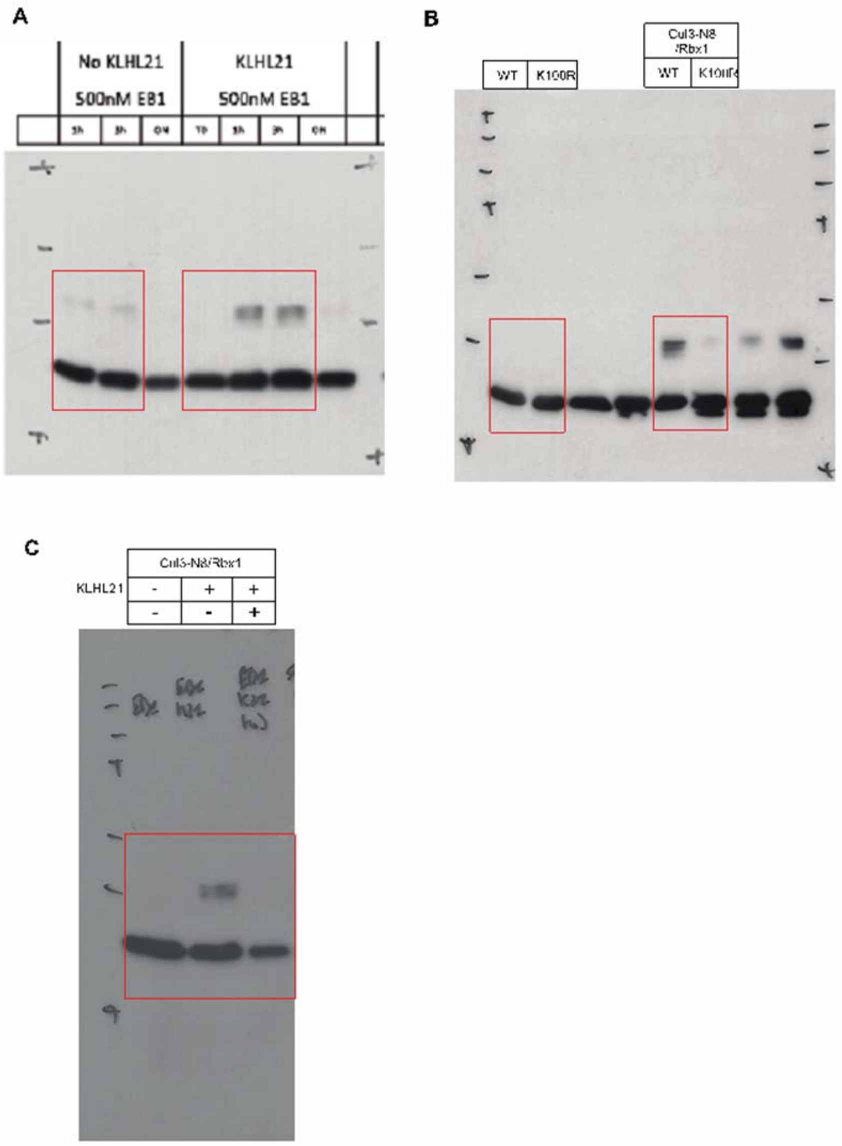

Supplementary Figure 7:
(A), (B), (C) full blots of respectively fig. 3B, fig. 3E, fig. 3F

SUPPLEMENTARY TABLE

protein name	Uniprot acc. number	protein probability	peptide sequence	nsp adjusted probability	spectral counts	K position
EB1/MARE1	Q15691	1	IEQLCSGAAYCQFMDMLFPGSIALKK	0,9791	2	60
EB1/MARE1	Q15691	1	FQAKLEHEYIQNFK	0,9993	1	66
EB1/MARE1	Q15691	1	MGVDKIIPVDK	0,9993	1	89
EB1/MARE1	Q15691	1	KFQDNFEFVQWFK	0,9997	4	100
EB1/MARE1	Q15691	1	GKFQDNFEFVQWFK	0,9994	3	100
EB1/MARE1	Q15691	1	FQDNFEFVQWFK	0,9995	2	112
EB1/MARE1	Q15691	1	FFDANYDGKDYDPVAAR	0,9997	1	122
EB1/MARE1	Q15691	1	KPLTSSSAAPQRPISTQR	0,9997	2	151
EB1/MARE1	Q15691	1	QGQETAVAPSLVAPALNKPKKPLTSSSAAPQRPISTQR	0,994	1	151
EB1/MARE1	Q15691	1	QGQETAVAPSLVAPALNKPK	0,9948	2	150
EB1/MARE1	Q15691	1	QGQETAVAPSLVAPALNKPK	0,9908	2	150
EB1/MARE1	Q15691	1	TAAAPKAGPGVVR	0,996	1	174
EB1/MARE1	Q15691	1	KNPGVGNGDDEAAELMQQVNVLK	0,9997	4	182
EB1/MARE1	Q15691	1	DFYFGKLR	0,9017	1	220

Supplementary Table 1: TABLE 1: Mass-spectrometry analysis of EB1 peptides ubiquitylated by reconstituted CRL3^{KLHL21} complexes *in vitro*

Identification of ubiquitylated lysine (K) residues in human EB1 (Uniprot accession number Q15691). Human EB1 was ubiquitylated *in vitro* with reconstituted CRL3^{KLHL21} complexes and analysed by LC-MS/MS. Peptide sequence, spectral counting and protein probability of EB1 tryptic peptides containing a GlyGly-tag mass shift corresponding to ubiquitylated residues (in bold and underlined). The position of the modified lysine (K) in human EB1 is indicated in the last column.

SUPPLEMENTARY METHODS

Recombinant protein expression and purification

Cloning, recombinant baculovirus generation, expression and purification of human full-length Cul3/Rbx1, as well as production of wild type human Nedd8 with native N- and C-termini were performed as described in[44]. Neddylation and purification of neddylated Cul3/Rbx1 was performed as previously described for Cul1/Rbx1[45].

Full-length wild type human EB1 was cloned with a PreScission cleavable N-terminal StrepII2x-tag in a pET17b expression vector. Expression and affinity purification proceeded as described for Nedd8 above. The d-desthiobiotin eluted EB1 was incubated in a 1:50 molar ratio with GST-tagged PreScisscion protease at 4 C overnight to remove the StrepII^{2x}-tag. EB1 was further purified by size exclusion chromatography over a Superdex75 column, fitted with a Strep-Tactin Cartridge trap, buffer exchanging into 150 mM NaCl, 20 mM Hepes, pH 7.6, 2% glycerol, 2 mM DTT.

Full-length wild type human KLHL21 was cloned in a pGEX-5 expression vector and expressed and cells were lysed as described for Nedd8 above. The soluble supernatant was bound to a 5 ml GST HiTrap column (GE Healthcare) with a flow rate of 1 ml/min. The column was washed with 20 column volumes (CV) of washing buffer and eluted with 5 CV washing buffer, the salt concentration was reduced to 200 mM NaCl, and supplemented with 10 mM reduced L-gluthatione (AppliChem). The eluted GST-Klhl21 was incubated in a 1:50 molar ratio with GST-tagged PreScisscion protease at 4 C and buffer exchanged by dialysis into 150 mM NaCl, 20 mM Hepes, pH 7.6,

10% glycerol, 2 mM DTT. To purify the cleaved GST and GST-PreScission, the reaction was run through a GST HiTrap column a second time, and the Klhl21 containing flow through was collected.

EB1 mutant constructs were synthesized (GenScript) and N-terminally 6xHIS-tagged EB1 mutants were expressed in BL21 overnight at 18°C after induction with 0.1 mM IPTG. For purification of individual EB1 mutants the lysis buffer (25 mM Na_2HPO_4/NaH_2PO_4 pH 7.0, 400 mM NaCl, 20 mM imidazole) was supplemented with PMSF, complete protease inhibitor tablets (Roche) and lysozyme (0.1 mg/ml). The cells were lysed by sonication, pre-cleared by centrifugation and incubated with Ni-NTA Agarose (QIAGEN) for 3 h at 4°C. The proteins were eluted from the beads with elution buffer (25 mM Na_2HPO4/NaH_2PO_4 pH 7.0, 200 mM imidazole and 150 mM NaCl) and further purified over a strong ion-exchange column (HiTrap Q 1 ml, GE Healthcare, 150 mM to 1 M NaCl salt gradient). Peak fractions were pooled, subsequently transferred into the storage buffer (25 mM Na_2HPO_4/NaH_2PO_4 pH 7.0, 150 mM NaCl, 5% glycerol) via PD-10 desalting columns (GE Healthcare) and snap-frozen at -80°C.

Tryptic digestion and mass-spectrometry analysis

Proteins were reduced with 12.5 mM dithiotreitol for 30 min at 32°C and alkylated with 40 mM iodoacetamide for 45 min at 25 °C, in the dark. Samples were diluted with 0.1 M NH_4HCO_3 to a final concentration of 1.5 M urea and incubated overnight at 32°C with sequencing-grade porcine trypsin (Promega, Madison, WI, USA) added to a final enzyme:substrate ratio of 1:100. The digestion was stopped by acidification with formic acid to a final pH < 3, the peptide mixtures desalted on Sep-Pak tC18 cartridges (Waters,

Milford, MA, USA) and eluted with 80% acetonitrile. All peptide samples were evaporated on a vacuum centrifuge to dryness, resolubilised in 0.1% formic acid and immediately analysed on a Triple-TOF mass spectrometer (5600 QTrap, ABSciex) equipped with a nano-electrospray ion source. Peptides were loaded and chromatographically separated using a linear gradient from 2 to 35% acetonitrile in 140 min and a flow rate of 300 nl/min. The raw data (Wiff files) were converted in to mzXML files using MS data converter (ABSciex). Collected spectra were searched against the *Homo sapiens* protein database with Sorcerer™-SEQUEST® (Thermo Electron, San Jose, CA, USA). Trypsin was set as the digesting protease with the tolerance of two missed cleavages, one non-tryptic terminus and not allowing for cleavages of KP and RP peptide bonds. The monoisotopic peptide and fragment mass tolerances were set to 50 ppm and 0.8 Da respectively. Carboxyamidomethylation of cysteines (+57.0214 Da) was defined as fixed modification and oxidation of methionines (+15.99492 Da) and di-glycine (GG) modification of lysines (+114.043 Da) as variable modifications. Protein identifications were statistically analysed with ProteinProphet (v3.0) and filtered to a cut-off of 0.9 ProteinProphet probability, which in this particular case corresponds to a FDR < 1%, calculated based on a target-decoy approach[46].
